# Nuclear actin regulates cell proliferation and migration via inhibition of SRF and TEAD

**DOI:** 10.1016/j.bbamcr.2020.118691

**Published:** 2020-07

**Authors:** Madeleine C. McNeill, Jason Wray, Graciela B. Sala-Newby, Charles C.T. Hindmarch, Sarah A. Smith, Reza Ebrahimighaei, Andrew C. Newby, Mark Bond

**Affiliations:** a School of Translational Health Sciences, Faculty of Health Sciences, University of Bristol, Research Floor Level 7, Bristol Royal Infirmary, Bristol BS2 8HW, UK; b Queen’s Cardiopulmonary Unit (QCPU), Translational Institute of Medicine (TIME), Department of Medicine, Queen’s University, Kingston, ON K7L3N6, Canada

**Keywords:** Actin, Nuclear, cAMP, Proliferation, Migration, YAP, TEAD, SRF

## Abstract

Actin dynamics regulate cell behaviour in response to physiological signals. Here we demonstrate a novel role for nuclear actin in inhibiting cell proliferation and migration. We demonstrate that physiological signals that elevate cAMP, which is anti-mitogenic in vascular smooth muscle cells, increases nuclear actin monomer levels. Expression of a nuclear-targeted polymerisation-defective actin mutant (NLS-Actin_R62D_) inhibited proliferation and migration. Preventing nuclear actin monomer accumulation by enhancing its nuclear export or polymerisation reversed the anti-mitogenic and anti-migratory effects of cAMP. Transcriptomic analysis identified repression of proliferation and migration associated genes regulated by serum response factor (SRF) and TEA Domain (TEAD) transcription factors. Accordingly, NLS-Actin_R62D_ inhibited SRF and TEAD activity and target gene expression, and these effects were reversed by constitutively-active mutants of the TEAD and SRF co-factors YAP, TAZ and MKL1. In summary, intranuclear actin inhibits proliferation and migration by inhibiting YAP-TEAD and MKL-SRF activity. This mechanism explains the anti-mitogenic and anti-migratory properties of physiological signals that elevate cAMP.

**Summary:**

McNeill et al show that increased levels of intranuclear actin monomer inhibit cell proliferation and migration by inhibiting MKL1-SRF and YAP/TAZ-TEAD-dependent gene expression. This mechanism mediates the anti-mitogenic and anti-migratory effects of physiological signals that elevate cyclic-AMP.

## Highlights

• Elevated cAMP increases levels of nuclear actin in vascular smooth muscle cells.
• Nuclear actin inhibits vascular smooth muscle cell proliferation and migration.
• Nuclear actin mediates anti-mitogenic and anti-migratory properties of cAMP.
• TEAD and SRF inhibition mediates growth inhibitory effects of nuclear actin.
• TEAD and SRF inhibition mediates inhibition of migration by nuclear actin.

## 1 Introduction

Most cells respond to a complex multitude of mechanical and biochemical, micro-environmental signals by reorganising their actin cytoskeleton. This plays a central role in determining appropriate cellular responses including proliferation and migration. For example, cell adhesion to specific extracellular matrix (ECM) proteins influences cell proliferation via integrin dependent changes in cell shape and actin cytoskeleton organisation [1,6] Generation of tensile forces in F-actin stress fibres in response to changes in ECM stiffness, cell spreading, or cell-to-cell contact are also essential for cell-cycle progression in adhesion-dependent cell types [13,47]. Likewise, actin cytoskeleton polymerisation and formation of tensed F-actin stress fibres are essential for cell cycle progression in response to mitogen stimulation [21]. Pharmacological disruption of actin polymerisation with specific actin binding drugs (e.g. cytochalasin-D or latrunculin-B) or by imposing constraints on cell spreading blocks the ability of cells to respond to mitogens and proliferate [4,29,30]. Importantly, reduced actin polymerisation and stress fibre formation has also been implicated in the anti-mitogenic actions of physiological signals, including activators of G_s_ coupled GPCRs that elevate intracellular levels of 3′,5′-cyclic adenosine monophosphate (cAMP) [5,24,35,36,53]. In some cell types, including vascular smooth muscle cells (VSMC), elevated cAMP potently blocks cell proliferation and migration [[26], [27], [28],^55^]. In these cells, the anti-mitogenic and anti-migratory properties of cAMP are dependent on inhibition of RhoGTPase activity, including RhoA and Rac1, resulting in impaired actin polymerisation [5]. For example, expression of constitutively active mutants of RhoA or Rac1 prevents cAMP induced disruption of actin stress fibres and reverses growth arrest [5]. These observations suggest that the actin cytoskeleton acts as an important point of convergence of multiple growth regulatory pathways and signals from the local microenvironment to control proliferation and migration.

Several mechanisms have been proposed to explain how actin cytoskeleton reorganisation controls cell fate. For example, cell cycle arrest in response to cytoskeleton disruption is associated with failure to induce sustained MAPK activity in response to mitogen stimulation [49], which prevents induction of Cyclin D and downregulation the cyclin-dependent kinase inhibitor p27^KIP1^ [25]. Together this prevents cyclin dependent kinases activation and G_1_-restriction point transit. However, the anti-mitogenic effects of signals that disrupt the actin cytoskeleton can be dissociated from their effects on MAPK activity and cyclin expression [7,25]. For example, disruption of actin polymerisation in response to cAMP elevating stimuli inhibits S phase entry in cells in which MEK activity has been pharmacologically inhibited [7], thus, implicating additional early regulatory mechanisms.

Most research on actin has focussed on understanding its function as a component of the cytoplasmic cytoskeleton. However, several reports provide evidence that actin is present within the nucleus [8,31,61], where it plays a significant role in several nuclear processes, including transcription and chromatin remodelling. Early studies detected actin or actin-like proteins in the nuclei of various cells [11,31,38,44]. However, the evidence was not widely accepted and believed to be a contamination artefact. Furthermore, the mere presence of actin in nuclei was not considered strong evidence that it has a role in nuclear functions. Only recently has the presence of actin within the nucleus become widely accepted and recognised to play important roles in nuclear processes, including RNA polymerase activity, gene transcription, heterochromatin remodelling and regulation of cell fate decisions, including apoptosis and differentiation [32].

Levels of nuclear actin are actively controlled and highly regulated. Translocation of actin, which lacks an intrinsic nuclear localisation signal, into the nucleus depends on interaction with cofilin. These actin:cofilin complexes are transported into the nucleus via interaction with the specific importer protein Importin-9 (IPO9). Although β-actin contains two nuclear export sequences, actin nuclear export is dependent on interaction with exportin-6 (XPO6) in association with Profilin. Actin in the nucleus can rapidly transition between monomeric and polymeric forms. Actin polymerisation within the nucleus can be promoted by members of the diaphanous-related formins such as mDIA1 and mDIA2 and members of the RhoGTPases, such as RhoA, which have both been detected within the nucleus [32,58]. This high degree of regulation is consistent with the important functional roles for intranuclear actin [32,50,58].

Although the presence of actin within the nucleus and its importance in chromatin and transcriptional regulation is now widely accepted, relatively little is known about the physiological signals that modulate nuclear actin levels and the importance of this in the regulation of cell behaviour. Based on observations from our laboratory that the anti-mitogenic and anti-migratory properties of elevated cAMP are dependent remodelling of the actin cytoskeleton [5,24,35,36,53], we hypothesised that cAMP elevating stimuli would modulate levels of intra-nuclear actin and that this regulation of intranuclear actin level might underlie the anti-mitogenic and anti-migratory effects of cAMP.

## 2 Results

### 2.1 Cyclic-AMP elevating stimuli increase levels of nuclear actin monomer

**Fig. 1:**
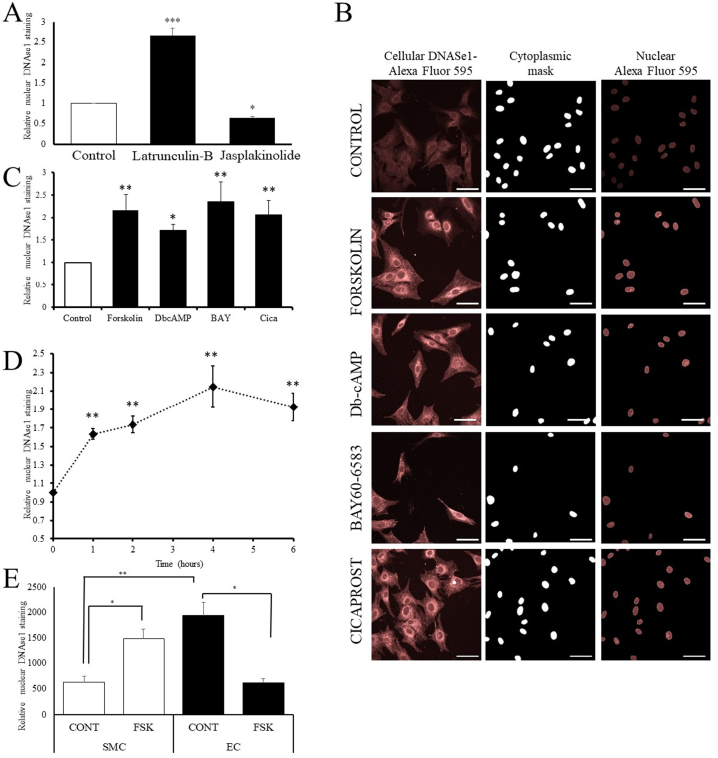
cAMP signalling increases levels of intranuclear actin VSMCs were stimulated with Latrunculin-B (0.25 μg/ml) or Jasplakinolide (0.5 μM) for 2 h and nuclear actin monomer quantified by DNAse1 staining (A). VSMC were stimulated forskolin (25 μM), Db-cAMP (200 μM), BAY65-60583 (BAY; 1 μg/ml) or Cicaprost (Cica; 2 μM) for 2 h and stained for actin monomer with DNAse1 (B) and intranuclear actin levels quantified (C). Cytoplasmic mask represents a black and white image generated from the DAPI nuclear signal and used to mask the cytoplasmic DNAse1 signal. Scale bar indicates 50 μm. Cells were stimulated with BAY60-6583 (1 μg/ml) for indicated times and intranuclear actin levels quantified (D). VSMCs and HUVECs were stimulated with forskolin (25 μM) for 2 h and intranuclear actin levels quantified (E). Data is mean ± standard error. One-way ANOVA with Student Newman Keuls post-test. * indicates p < 0.05, ** indicates p < 0.01 and *** indicates p < 0.001.

We first tested if physiological anti-mitogenic signals modulate levels of nuclear actin monomer. We studied signals that elevate levels of the second messenger cAMP, since these have anti-mitogenic effects in several different cell types, including vascular smooth muscle cells [26,34,48], and have been linked to actin cytoskeleton remodelling [5,24]. We exploited the well characterised high affinity of DNAse1 for actin monomer over actin polymer [42] to detect and quantify intranuclear actin monomer levels, as previously described [37,57]. To validate this technique, we treated cells with latrunculin-b, to induce actin depolymerisation, or jasplakinolide, to induce actin polymerisation. Quantification of confocal z-stack images of DNAse1-Alexa Fluor-594 stained cells revealed that latrunculin-B significantly increased intranuclear staining of actin monomer and jasplakinolide significantly decreased nuclear staining of actin-monomer, consistent with specific detection of actin monomer (Fig. 1A). Furthermore, consistent with previous reports that cAMP induces depolymerisation of the cytoplasmic actin cytoskeleton in VSMCs [24], we observed an increase in cytoplasmic DNAse1 staining intensity in cells treated with the adenylate cyclase agonist, forskolin; the cAMP-analogue, dibutyryl-cAMP; the adenosine A2B-receptor agonist, BAY60-6583; or the prostacyclin, mimetic, Cicaprost (Fig. 1B). Importantly, all these stimuli also induced a significant increase in nuclear actin monomer levels (Fig. 1B and C). Similar increases in nuclear actin monomer levels were detected in human VSMCs in response to forskolin stimulation (Supplement Fig. 1). Time course analysis of BAY60-6583 stimulated cells demonstrated that nuclear actin monomer levels were significantly increased after one hour and were maximal after 4 h (Fig. 1D). By contrast, forskolin stimulation did not increase nuclear actin monomer levels in endothelial cells (ECs; Fig. 1E), where elevated cAMP signalling is not associated with growth arrest [53]. Taken together, these data demonstrate that physiological stimuli that elevate intracellular cAMP increase nuclear actin monomer levels in VSMCs but not ECs and that this corresponds with the previously described anti-mitogenic properties of cAMP in these two cell types [53].

### 2.2 Intranuclear actin monomer inhibits cell proliferation and migration in VSMCs

**Fig. 2:**
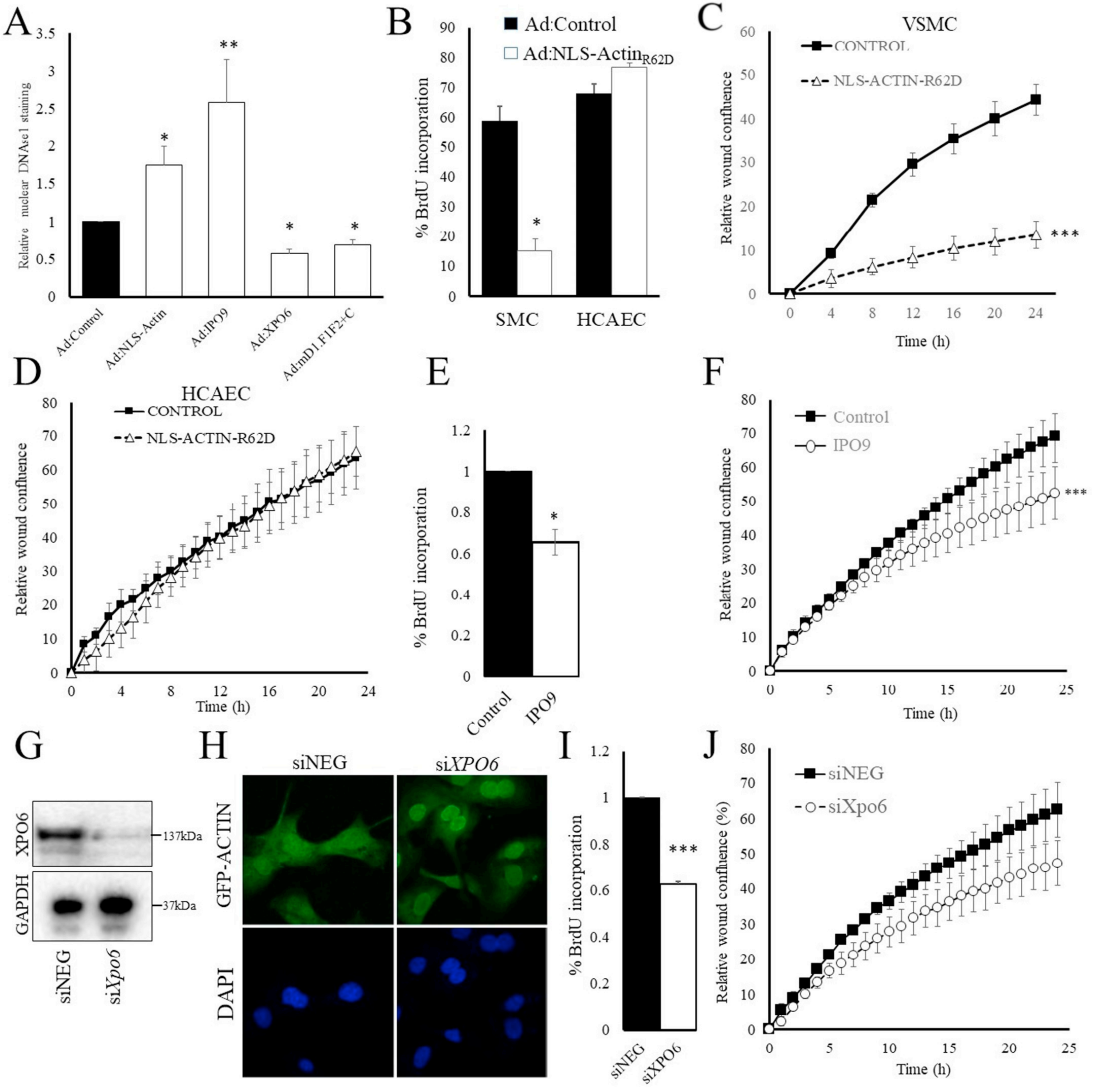
Intranuclear actin inhibits VSMC but not EC proliferation and migration. VSMCs were infected with either Ad:Control, Ad:NLS-Actin_R62D_ or Ad:IPO9, as indicated. Intranuclear actin levels were quantified by DNAse1 staining (A). VSMCs and HCAECs were infected with Ad:Control or Ad:NLS-Actin_R62D._ Cell proliferation was quantified by incorporation of Edu 24 h post infection (B). VSMC (C) and HCAEC (D) migration was quantified by Incucyte migration assay 24 h post infection. VSMC were infected with either Ad:Control or Ad:IPO9 and incorporation of Edu (E) and migration (F) quantified. VSMCs were transfected with siRNA targeting XPO6 (siXPO6). XPO6 and GAPDH protein levels were quantified 24 h post transfection by Western blotting (G). Intranuclear actin levels were quantified by co-transfection with GFP-Actin (H). Cell proliferation was quantified by incorporation of Edu (I) and cell migration quantified by Incucyte migration assay (J).

We next asked whether nuclear actin monomer not only correlates with but also plays a role in inhibiting VSMC proliferation and cell migration. We employed multiple approaches to specifically modulate nuclear actin monomer levels, including adenovirus mediated over expression of polymerisation defective mutant (R62D) of actin tagged with a nuclear localisation signal (Ad:NLS-Actin_R62D_); overexpression of the actin importer, importin 9 (Ad:IPO9); over expression of the actin exporter, XPO6 (Ad:XPO6); siRNA-mediated silencing of XPO6 (siXPO6); and over expression of a constitutively active mutant of mDIA1 (Ad:mDIA1-CT), which accumulates in the nucleus due to a cryptic nuclear localization sequence (NLS) [2]. Expression of NLS-Actin_R62D_ (Ad:NLS-Actin_R62D_) resulted in specific nuclear expression of FLAG-tagged Actin_R62D_ (Supplement Fig. 2). It is important to note that infection with Ad:NLS-Actin_R62D_ did not result in a detectable difference in the total cellular levels of β-actin, even though FLAG-tagged NLS-Actin_R62D_ was clearly detectable (Supplement Fig. 2B), indicating that expression from this vector is sufficient to specifically increase nuclear actin monomer levels without significantly affecting the total cellular pool of β-actin However, DNAse1-Alexa Fluor-594 staining detected a significant increase in actin monomer levels in cells infected with Ad:NLS-Actin_R62D_ (Fig. 2A). Infection of cells with Ad:IPO9 also significantly increased nuclear actin monomer levels (Fig. 2A), while infection with Ad:XPO6 or Ad:mDIA-CT significantly decrease basal nuclear actin monomer levels (Fig. 2A). Overexpression of mDIA-CT in cells expressing nuclear localised Lifeact-EGFP resulted in formation of highly organised nuclear actin network, suggesting that mDIA-CT reduced nuclear actin monomer levels by promoting nuclear actin polymerisation (Supplement Fig. 3).

Expression of NLS-Actin_R62D_ significantly inhibited proliferation of VSMCs (Fig. 2B and Supplement Fig. 3A) but not coronary artery endothelial cells (HCAECs) (Fig. 2B). Expression of NLS-Actin_R62D_ also significantly inhibited migration of VSMCs (Fig. 2C and Supplement Fig. 4B) but not HCAECs (Fig. 2D) in real-time scratch wound assays. To test the role of nuclear actin monomer in the regulation of proliferation and migration further, we increased nuclear actin levels by over expressing IPO9 and silencing XPO6. Infection of VSMCs with Ad:IPO9 significantly inhibited proliferation of VSMCs (Fig. 2E) and significantly inhibited their migration (Fig. 2F). Furthermore, transfection of cells with siRNA targeting XPO6 reduced XPO6 protein levels (Fig. 2G), increased levels of nuclear actin monomer (Fig. 2H), and significantly inhibited cell proliferation (Fig. 2I) and migration (Fig. 2J). Taken together, these data demonstrate that increased nuclear actin inhibits VSMC proliferation and migration.

### 2.3 Increased intranuclear actin monomer mediates that anti-mitogenic and anti-migratory effects of cAMP in VMSCs

**Fig. 3:**
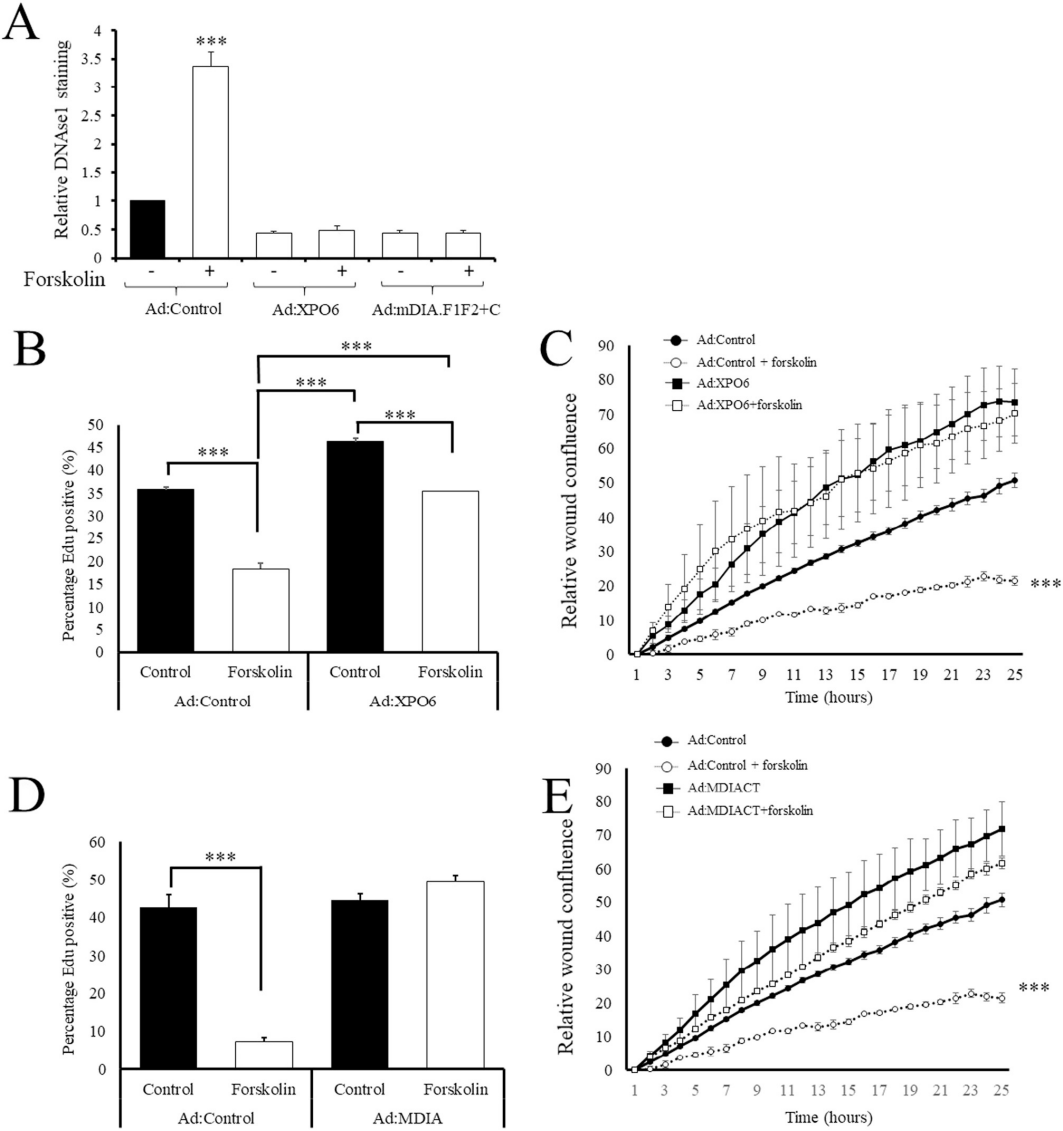
Increased intranuclear actin mediates the anti-proliferative and anti-migratory effects of cAMP in VSMCs VSMC were infected with either Ad:Control, Ad:XPO6 or Ad:mDIACT, as indicated. Cells were stimulated with 25 μM forskolin for 2 h and levels of intranuclear actin quantified by DNAse1 staining (A). VSMCs were infected with Ad:XPO6 (B and C) or Ad:mDIACT (D and E). Cell proliferation was quantified by incorporation of Edu (B and D). Cell migration was quantified by real time migration assay (C and E).

We went on to investigate to what extent the anti-mitogenic and anti-migratory effects of cAMP in VSMCs are dependent on increased levels of nuclear actin monomer. We used adenoviral mediated expression of XPO6 (Ad:XPO6) or mDIAF1F2+C (Ad:mDIA-CT) to reverse cAMP-mediated increases in nuclear actin monomer levels. Foskolin treatment of cells infected with a control adenovirus significantly increased nuclear actin monomer. However, infection with Ad:XPO6 or Ad:mDIA-CT significantly reduced basal nuclear actin monomer levels (Fig. 3A) and, importantly, completely reversed the forskolin induced increase in nuclear actin monomer levels, detected by DNAse1-Alexa Fluor-594 staining. Forskolin treatment of cells infected with a control adenovirus significantly inhibited cell proliferation (Fig. 3B and D), consistent with our previous observations [53]. Infection with Ad:XPO6, however, significantly increased basal proliferation rates and rescued proliferation after forskolin treatment to levels that were not significantly different from untreated Ad:Control infected cells (Fig. 3B). Ad:XPO6 infection also significantly increased basal migration rates and completely prevented the inhibitory effects of forskolin on migration (Fig. 3C). Overexpression of XPO6 also reversed the anti-mitogenic (Supplement Fig. 5A and B) and anti-migratory effects (Supplement Fig. 5C and D) of the adenosine A2B-receptor agonist BAY60-6582. Infection with Ad:mDIA-CT, also completely reversed forskolin mediated cell-cycle arrest (Fig. 3D), without affecting basal proliferation rates. Infection with Ad:mDIA-CT significantly increase basal migration rates (Fig. 3E). Although forskolin treatment resulted in a small but significant inhibition of migration, migration rates in these cells was still significantly higher than that in untreated Ad:Control infected cells (Fig. 3E). Taken together, these data demonstrate that cAMP-elevating stimuli increase levels of intranuclear actin monomer and that this is essential for the anti-mitogenic and anti-migratory properties of cAMP in VSMCs.

### 2.4 Transcriptomic analysis reveals that intranuclear actin and elevated cAMP repress SRF and TEAD target genes associated with proliferation and migration

**Fig. 4:**
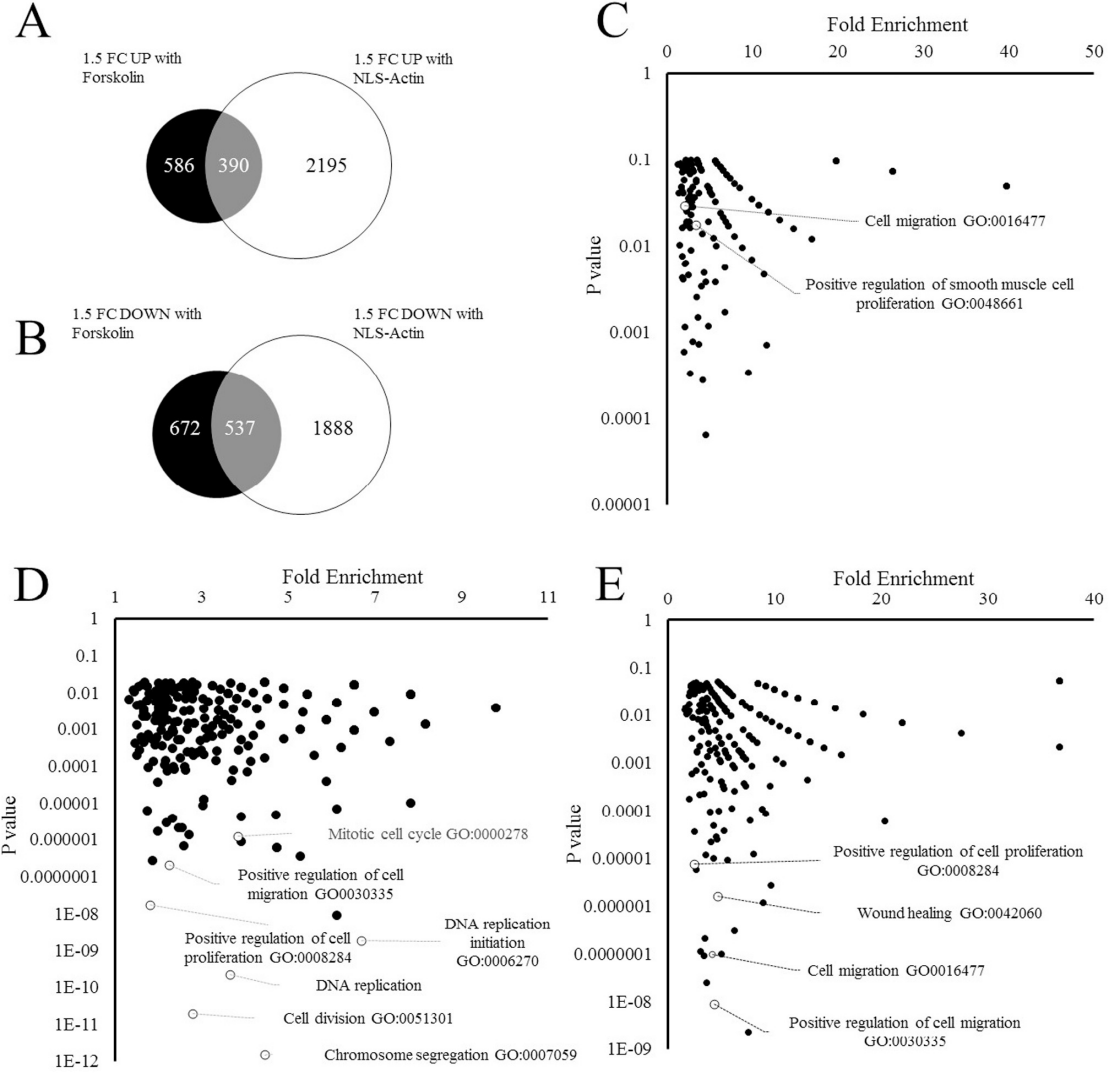
Transcriptomic analysis of nuclear actin and cAMP regulated genes Venn diagrams of genes upregulated (A) and down regulated (B) in response to a 6-h stimulation with 25 μM forskolin (black circles) or expression of NLS-Actin_R62D_ (white circles). Gene ontology analysis of genes down regulated >1.5 fold in response to 6-h forskolin stimulation (C); Genes down regulated >1.5 fold in response NLS-Actin_R62D_ (D); or gene down regulated by >1.5 fold by 25 μM forskolin or NLS-Actin_R62D_ (E).

We performed RNA-seq to compare the transcriptomic profile of cells treated with forskolin or expressing NLS-Actin_R62D_ to gain an insight into the mechanisms underlying the anti-mitogenic effects of nuclear actin (see supplementary data file S1). Forskolin treatment significantly increased the expression of 976 genes by >1.5 fold and decrease the expression of 1209 genes by >1.5 fold compared to controls. Expression of NLS-Actin_R62D_ increased the expression of 2585 genes by >1.5 fold and decrease the expression of 2425 genes by >1.5 fold compared to Ad:Control (Fig. 4A and B). Of these genes, 390 were upregulated by both forskolin and NLS-Actin_R62D_ and 537 were downregulated by forskolin and NLS-Actin_R62D_. Gene ontology (GO) enrichment analysis of genes downregulated >1.5 fold by forskolin identified significant enrichment of genes associated with positive regulation of smooth muscle cell proliferation (GO:0048661) and cell migration (GO:0016477) (Fig. 4C). Likewise, GO enrichment analysis of genes downregulated >1.5 fold by NLS-Actin_R62D_ also identified significant enrichment of genes associated with positive regulation of cell migration (GO:0016477), DNA replication (GO:0006260), and positive regulation of cell proliferation (GO:0008284) (Fig. 4D). Importantly, the 537 genes that were down regulated by forskolin or NLS-Actin_R62D_ were also enriched in genes associated with also positive regulation of cell proliferation (GO:0008284) and cell migration (GO:0030334) (Fig. 4E). Taken together, these data indicate that cAMP and intranuclear actin monomer regulate a common set of genes associated with proliferation and cell migration.

**Fig. 5:**
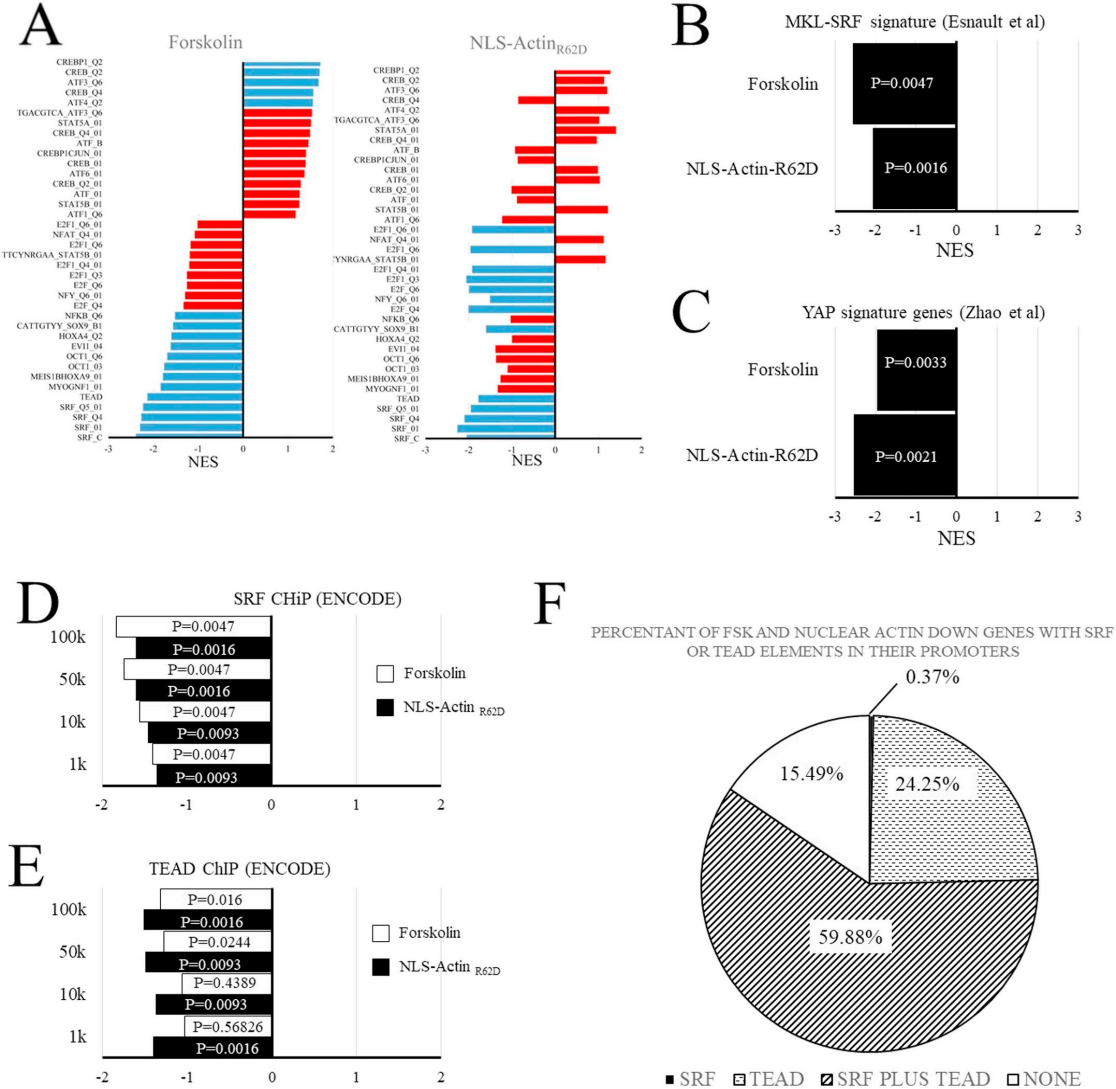
Gene Set Enrichment Analysis (GSEA) of intranuclear actin and forskolin regulated genes. GSEA of broad institute molecular signatures database C3:motif gene sets in NLS-Actin_R62D_ and forskolin regulated genes (A). GSEA of an MKL-SRF gene signature gene set [17] in NLS-Actin_R62D_ and forskolin regulated genes (B). GSEA of a YAP gene signature gene set [70] in NLS-Actin_R62D_ and forskolin regulated genes (C). GSEA of genes with a SRF ChIP-seq peak (ENCODE) within 1 kbp, 10 kbp, 50 kbp or 100 kbp of their TSS (D). GSEA of genes with a TEAD4 ChIP-seq peak (ENCODE) within 1 kbp, 10 kbp, 50 kbp or 100 kbp of their TSS (D). GSEA of genes with a SRF ChIP-seq peak (ENCODE) within 1 kbp, 10 kbp, 50 kbp or 100 kbp of their TSS (E). Venn diagram showing percentage of genes that have either an SRF element (MA0083.1; MA0083.2;MA0083.3), a TEAD element (MA0090.1; MA0090.2; MA0808.1; MA0809.1) or both within 1 kbp upstream of the TSS, which are repressed >1.5 fold by NLS-Actin_R62D_ and forskolin (F).

We performed gene set enrichment analysis (GSEA) to identify upstream regulatory transcription factors potentially involved in mediating gene expression changes in response to cAMP or nuclear actin monomer. We initially searched for enrichment of Molecular Signatures Database (MSigDB; Broad Institute) transcription factor target gene sets in ranked lists of genes derived from the NLS-Actin_R62D_ and forskolin datasets. As expected, GSEA of the forskolin dataset identified enrichment of CREB and ATF3 target genes amongst the genes positively regulated by forskolin, consistent with well characterised activation of these transcription factors by cAMP (Fig. 5A). However, neither CREB nor ATF3 were enriched in the NLS-Actin_R62D_ gene set, implying selective regulation of these TFs by cAMP. Importantly, we identified significant enrichment of SRF and TEAD target genes amongst the genes negatively regulated by forskolin and NLS-Actin_R62D_ (Fig. 5A), suggesting that SRF and TEAD TFs are inhibited by elevated cAMP and by nuclear actin. To test this further, we searched for an enrichment of genes identified by Esnault et al [17] as MKL-SRF signature genes and genes identified by Zhao et al. [70] as upregulated by over expression the TEAD co-factor Yes Associated Protein-1 (YAP). This analysis identified significant enrichment of MKL-SRF signature genes (Fig. 5B) and enrichment of YAP-induced genes (Fig. 5C) amongst the genes repressed by forskolin and by NLS-Actin_R62D_, again indicating selective down regulation of MKL-SRF and YAP-target genes by cAMP and nuclear actin. Finally, we looked for enrichment of genes having an SRF or TEAD binding sites within 1kbp, 10kbp, 50kbp or 100 kbp of their TSS, identified by SRF or TEAD4 ChiP respectively (ENCODE database). This analysis identified significant enrichment of genes with at least one SRF or TEAD ChIP binding sites within the promoter regions of the NLS-Actin_R62D_ and the forskolin repressed gene sets (Fig. 5D and E). Finally, we searched for consensus SRF (Jaspar SRF motif MA0083.1) and TEAD elements (Jaspar TEAD motifs MA0090.1, MA0090.2, MA0808.1, MA0809.1, MA1121.1) within 10kbp upstream of the TSS of genes co-ordinately down regulated by forskolin of NLS-Actin_R62D_. We found that 59.88% of these genes contain at least one SRF and TEAD binding element; 24.25% of genes contained at least one TEAD element but no SRF elements and 0.37% of genes contained at least one SRF element but no TEAD element (Fig. 5F). We did not detect either SRF or TEAD elements in 15.49% of the genes.

### 2.5 Intranuclear actin inhibits SRF and TEAD activity and target gene expression

**Fig. 6:**
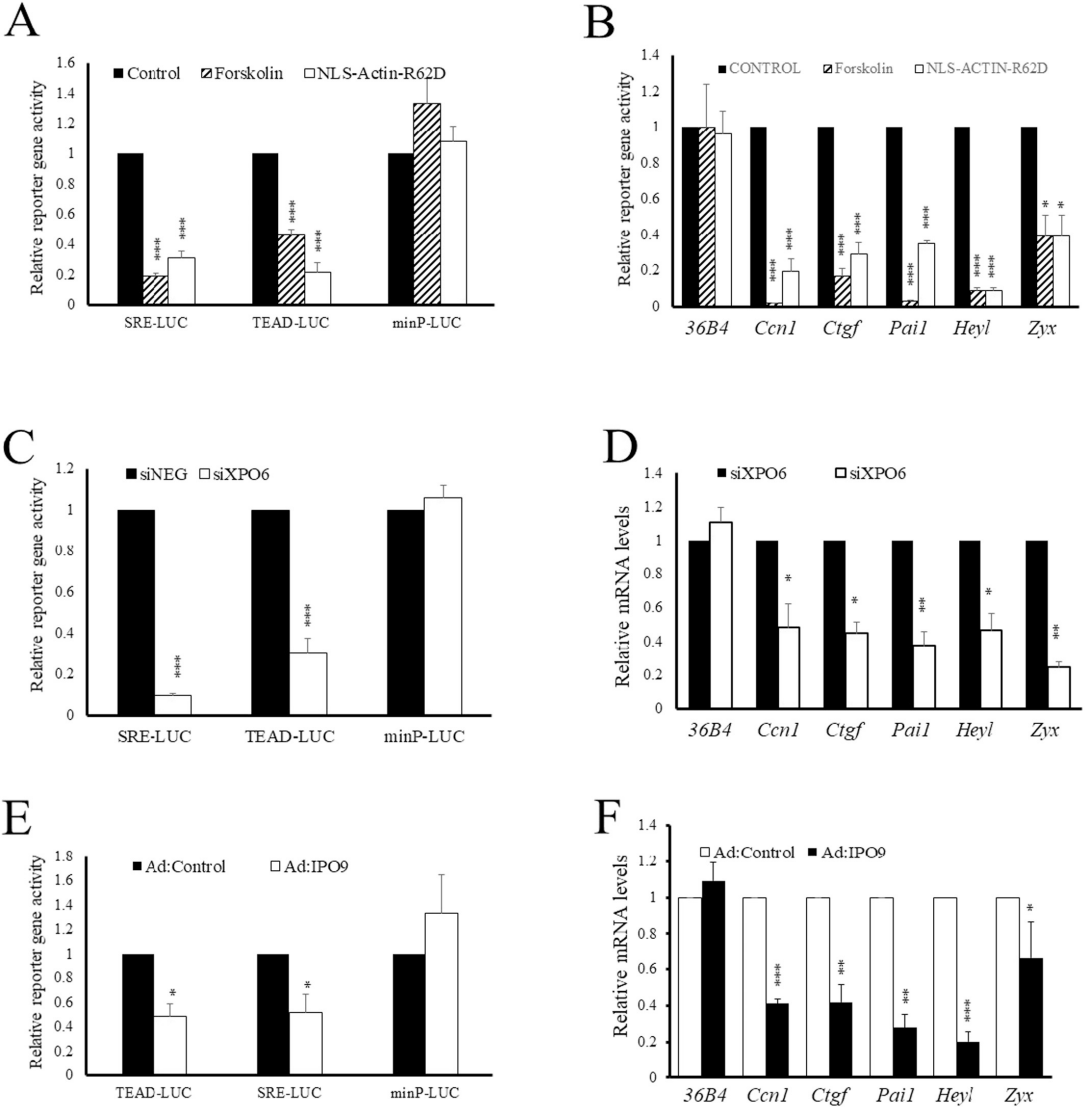
Intranuclear actin inhibits SRF and TEAD activity and target gene expression. VSMCs were stimulated with 25 μM forskolin for 6 h or infected with Ad:NLS-Actin_R62D_ as indicated. SRE-LUC or TEAD-LUC (A, C, E) activity was quantified. Total RNA was extracted and analysed using qRT-PCR for mRNA levels of SRF and TEAD target genes (B, D, F). Levels of the SRF and TEAD-independent housekeeping gene 36B4 was used as a control. * indicates p < 0.05, ** indicates p < 0.01, *** indicates p < 0.001.

Since SRF and TEAD TFs were identified by our GSEA and TF binding element enrichment analysis, we next asked if nuclear actin monomer inhibited their activity in VSMCs. As a positive control cells were treated with forskolin, which significantly inhibited SRE- and TEAD-dependent reporter gene activity (Fig. 6A), consistent with our previous findings [36,52]. Importantly, expression of NLS-Actin_R62D_ also significantly inhibited SRE- and TEAD-dependent reporter activity (Fig. 6A), without affecting activity of a reporter driven by a minimal promoter (minP-LUC) lacking SRE or TEAD elements. Forskolin and NLS-Actin_R62D_ also significantly inhibited steady-state mRNA levels of SRF- and TEAD-target genes, *Ccn1*, *Ctgf*, *Pai1, Heyl* and *Zyxin (Zyx)*, without affecting levels of the housekeeping gene *36B4* (Fig. 6B), consistent with specific inhibition of SRF and TEAD activity by intranuclear actin. To test this further, we prevented nuclear export of actin by silencing XPO6. This significantly inhibited SRF and TEAD-dependent reporter activity (Fig. 6C) and significantly inhibited mRNA levels of *Ccn1*, *Ctgf*, *Pai1*, *Heyl and Zyxin* without affecting the levels of the housekeeping gene *36B4* (Fig. 6D). Lastly, we increased nuclear actin levels by over expressing IPO9. This significantly inhibited SRE- and TEAD-dependent reporter activity, without affecting activity of a minimal promoter reporter (Fig. 6E) and significantly inhibited expression of the TEAD and SRF-target genes *Ccn1, Ctgf, Pai1, Heyl and Zyxin*. Taken together, these data demonstrate that increased intranuclear actin inhibits SRF and TEAD activity and target gene expression.

The transcriptional activity of TEAD is dependent on the transcriptional co-factors Yes-Associated Protein (YAP) [62]. In a similar manner, transcriptional activity of SRF can be regulated by the transcriptional co-factor MKL1 [43]. To better understand the mechanisms underlying the inhibition of SRF and TEAD we tested if nuclear actin affected the cellular localisation of YAP and MKL1, respectively. Expression of NLS-Actin_R62D_ did not significantly affect levels of nuclear or cytoplasmic YAP and hence the cytoplasmic:nuclear ratio was unchanged (Supplement Fig. 6A and B). However, stimulation with forskolin, which induces loss of cytoplasmic actin stress fibres [53], did induce a reduction in nuclear YAP and an increase in the cytoplasmic:nuclear YAP ratio (Fig. 4C and D), consistent with our previous data showing that elevated cAMP induces YAP nuclear export [36]. Expression of NLS-Actin_R62D_ completely prevented nuclear localisation of MKL1 (Fig. 4E and F).

We next asked in intranuclear actin modulated phosphorylation levels of YAP, since increased YAP phosphorylation is associated with reduced TEAD activity [67]. Although disruption of actin polymerisation with latrunculin-B strongly increased global YAP phosphorylation, detected by reduced electrophoretic mobility on Phostag-acrylamide gels (Supplement Fig. 7A and B), and phosphorylation on serine-397, expression of NLS-Actin_R62D_ did not change YAP phosphorylation globally or specifically on serine-127 or serine-397 (Supplement Fig. 7A and B). Taken together, these data indicated that intranuclear actin inhibits SRF by preventing nuclear accumulation of MKL1 but intranuclear actin mediated inhibition of TEAD occurs independently of changes in nuclear YAP levels or phosphorylation status.

### 2.6 Increased intranuclear actin mediates the inhibitory effects of cAMP on SRF and TEAD-dependent gene expression

**Fig. 7:**
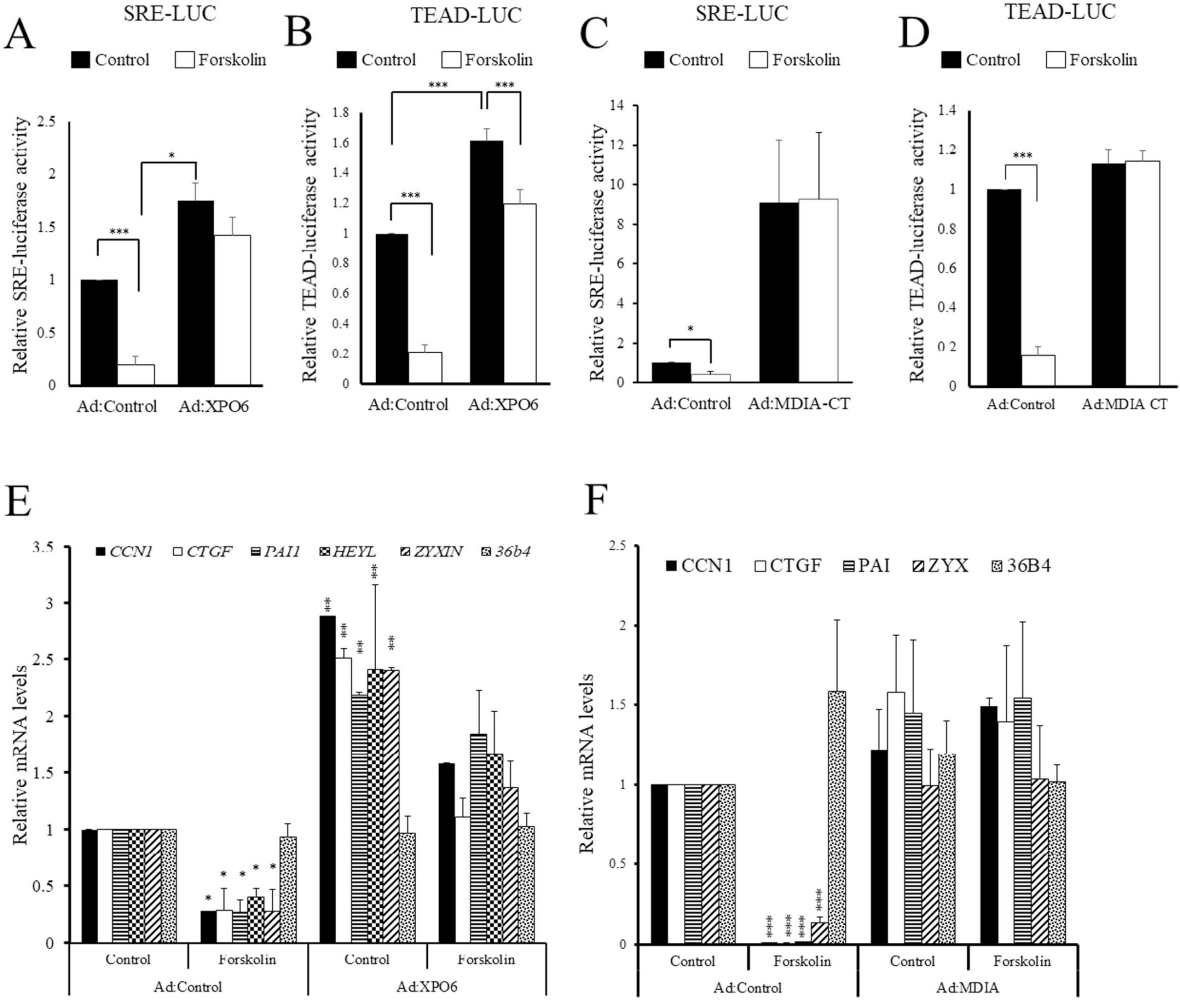
Increased nuclear actin mediates the inhibitory effects of cAMP on SRF and TEAD activity and target gene expression. VSMCs were transfected with SRE-LUC (A and C) or TEAD-LUC (B and D). Cells were infected with Ad:Control and either Ad:XPO6 (A and B) or Ad:mDIACT (C and D). Reporter gene activity was quantified 24 h post infection (A-D). VSMC were infected with Ad:XPO6 (E) or Ad:mDIACT (F). Total RNA was extracted 24 h post infection and analysed for SRF and TEAD-target genes using qRT-PCR (E and F).

We previously reported and confirmed here that cAMP signalling inhibits SRF and TEAD-dependent gene expression in VSMCs and that this is important in mediating the anti-mitogenic properties of cAMP in these cells [36,53]. Therefore, we tested if increased nuclear actin underlies these inhibitory effects of cAMP signalling on SRF and TEAD dependent gene expression. We over expressed of XPO6 or constitutively active nuclear mDIA1 (mDIA:F1F2+C) to reverse the forskolin induced increase in nuclear actin monomer and analysed effects on SRF and TEAD reporter activity and target gene expression. In control adenovirus infected cells forskolin significantly inhibited SRE-LUC (Fig. 7A and C) or TEAD-LUC (Fig. 7B and D) reporter activity. Over expression of XPO6 significantly increased basal activity of SRE-LUC (Fig. 7A) or TEAD-LUC (Fig. 7B). Importantly, expression XPO6 rescued SRE-LUC and TEAD-LUC activity to levels that were not different from unstimulated Ad:Control infected cells. Moreover, expression of mDIACT also completely reversed the forskolin-mediated inhibition of SRE-LUC (Fig. 7C) and TEAD-LUC (Fig. 7) activity. These data indicate that cAMP signalling inhibits SRF and TEAD activity, at least in part by elevating nuclear actin monomer levels. To test this further we analysed effects on SRF and TEAD-target gene mRNA levels. Over expression of XPO6 significantly increased basal expression of *Ccn1, Ctgf, Pai1, Heyl and Zyxin* and rescued expression after forskolin treatment to levels not different from unstimulated Ad:Control infected cells (Fig. 7E). In a similar way, expression of mDIACT completely prevented the forskolin mediated inhibition of *Ccn1, Ctgf, Pai1, Heyl and Zyxin* mRNA levels (Fig. 7F). Taken together, these data demonstrate that elevated cAMP inhibits SRF and TEAD-dependent gene expression at least in part by increasing intranuclear actin monomer levels.

### 2.7 Intranuclear actin mediated inhibition of TEAD activity is independent of effects on MKL1

Recent studies have reported mutual dependence of MKL-SRF and YAP-TEAD pathways [19]. Cross talk between MKL and YAP-TEAD activity has also been reported via formation of an MKL-YAP-TEAD ternary complex [33]. We therefore used over expression of constitutively active mutants of YAP (YAP_S27A_), TAZ (TAZ_5SA_) or MKL1 (MKL1_Δ100_) to test if inhibition of either TEAD or SRF activity by intranuclear actin was direct or else mediated via crosstalk between these two pathways. NLS-Actin_R62D_ expression inhibited SRE-LUC activity in control virus infected cells (Supplement Fig. 8A). Expression of MKL1_Δ100_ strongly enhanced the basal SRE-LUC activity and prevented inhibition by NLS-Actin_R62D,_ consistent with the well characterised function of MKL1 as an SRF cofactor. Surprisingly, expression of either YAP_S127A_ or TAZ_5SA_ enhanced basal SRF-LUC activity, although to a lower level than induced by MKL1_Δ100,_ implying that YAP and TAZ can enhance SRF activity, possibly via cross talk between YAP/TAZ-TEAD and SRF. YAP_S127A_ or TAZ_5SA_ also prevented the inhibition of SRF-LUC activity in response to NLS-Actin_R62D_ (Supplement Fig. 8A). This suggests that repression of SRF activity by intranuclear actin is at least in part mediated via this crosstalk with the YAP/TAZ-TEAD pathway. Expression of NLS-Actin_R62D_ also inhibited TEAD-LUC activity (Supplement Fig. 8B). Expression of either YAP_S127A_ or TAZ_5SA_ strongly enhanced basal activity and reversed the inhibitory effects of NLS-Actin_R62D_. Importantly, expression of MKL1_Δ100_ did not significantly enhance basal TEAD-LUC activity or reverse the inhibitory effects of NLS-Actin_R62D_, implying that MKL1-SRF signalling does not cross talk with the YAP/TAZ-TEAD pathway. Taken together, these data suggest that intranuclear actin mediated inhibition of TEAD activity occurs independently of effects on MKL-SRF. However, intranuclear actin-mediated inhibition of SRF involves crosstalk from the YAP/TAZ-TEAD pathway.

### 2.8 Active mutants of YAP, TAZ and MKL reverse the inhibitory effects of intranuclear actin on SRF and TEAD-dependent gene expression, proliferation and migration

**Fig. 8:**
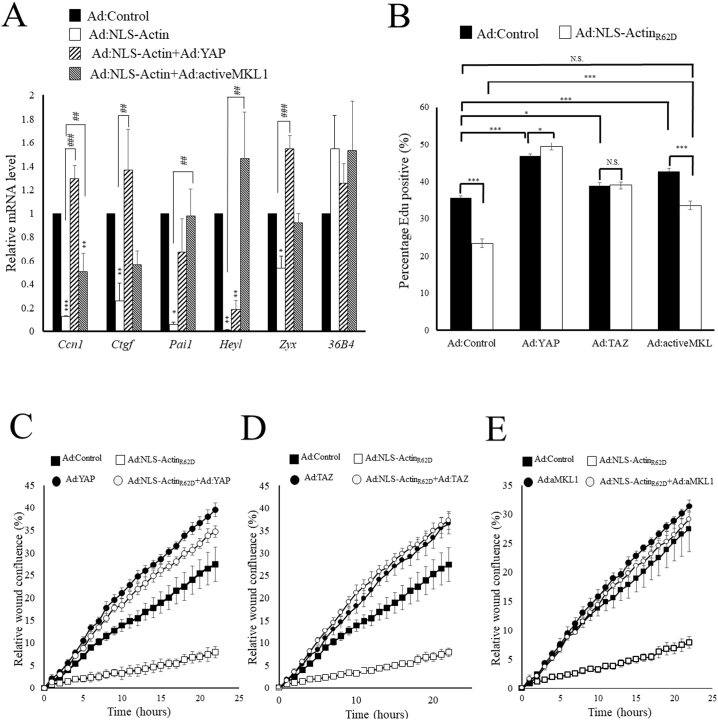
Active mutants of YAP, TAZ and MKL1 reverse the inhibitory effects of intranuclear actin on proliferation and migration VSMCs were infected with Ad:Control, Ad:NLS-Actin_R62D_, Ad:YAP, Ad:TAZ or Ad:activeMKL1 as indicated. Total RNA was extracted 24 h later and analysed by RT-PCR for the indicated target genes (A). One day post infection, cells were labelled with Edu for 4 h (B) or scratch wounded and migration quantified by Incucyte real time migration assay (C–E).

We next tested the importance of TEAD or SRF inhibition by nuclear actin in the regulation of endogenous gene expression, proliferation and migration. Expression of YAP_S127A_ significantly reversed the inhibitory effects of NLS-Actin_R62D_ on *Ccn1*, *Ctgf* and *Zyxin* mRNA levels but not levels of *Pai1* (Fig. 8A). Expression of MKL1_Δ100_ partially rescued mRNA levels of *Ccn1* and completely rescued levels of *Pai1, Heyl* and *Zyxin* but not *Ctgf* mRNA. This suggests that inhibition of TEAD an SRF co-factors, YAP and MKL1 respectively, are involved in mediating the inhibition of gene expression in response to increased nuclear actin. However, some genes display a preference for either YAP-TEAD (e.g. *Ctgf*) or MKL-SRF (e.g. *Heyl*). Expression of *Ccn1* and *Ctgf* have been previously reported to be required for efficient cell proliferation and migration [12,63]. We therefore tested if inhibition of *Heyl, Zyxin* and *Pai1* also contributes towards the growth inhibitory and anti-migratory effects of intranuclear actin. Transfection of cells with siRNA-targeting either *Heyl, Zyxin* or *Pai1* significantly reduced levels of the respective mRNAs (Supplement Fig. 9A, B and C) and significantly inhibited basal proliferation (Supplement Fig. 9D) and migration rates (Supplement Fig. 9E).

We tested the relative importance of MKL1-SRF and YAP/TAZ-TEAD inhibition for the growth inhibitory effects of intranuclear actin. Expression of NLS-Actin_R62D_ significantly inhibited cell proliferation (Fig. 8B), consistent with data presented in Fig. 2. Overexpression of YAP_S127A_ orTAZ_5SA_ significantly increased basal proliferation rates and completely abrogated the growth inhibitory effects of NLS-Actin_R62D_ (Fig. 8B). Expression MKL1_Δ100_ also significantly increased basal proliferation but only partially reversed the inhibitory effects of NLS-Actin_R62D_ (Fig. 8B). Expression of NLS-Actin_R62D_ significantly inhibited cell migration (Fig. 8C–E). Expression of either YAP_S127A_ or TAZ_5SA_ strongly enhanced basal migration rates above that of controls and completely reversed the inhibitory effects of NLS-Actin_R62D_, with migration rates in these cells remaining significantly higher than the basal migration rate of Ad:Control cells (Fig. 8C and D). Expression of MKL1_Δ100_ resulted in a small increase in basal migration rates and rescued the inhibitory effects of NLS-Actin_R62D_ to levels that were not different from basal rates in Ad:Control infected cells (Fig. 8E). Taken together, these data indicate that inhibition of TEAD is essential for the growth inhibitory and anti-migratory effects of nuclear actin monomer. Inhibition of SRF is also essential for the anti-migratory effects but only partially required for the growth inhibitory effects of nuclear actin.

## 3 Discussion

**Fig. 9:**
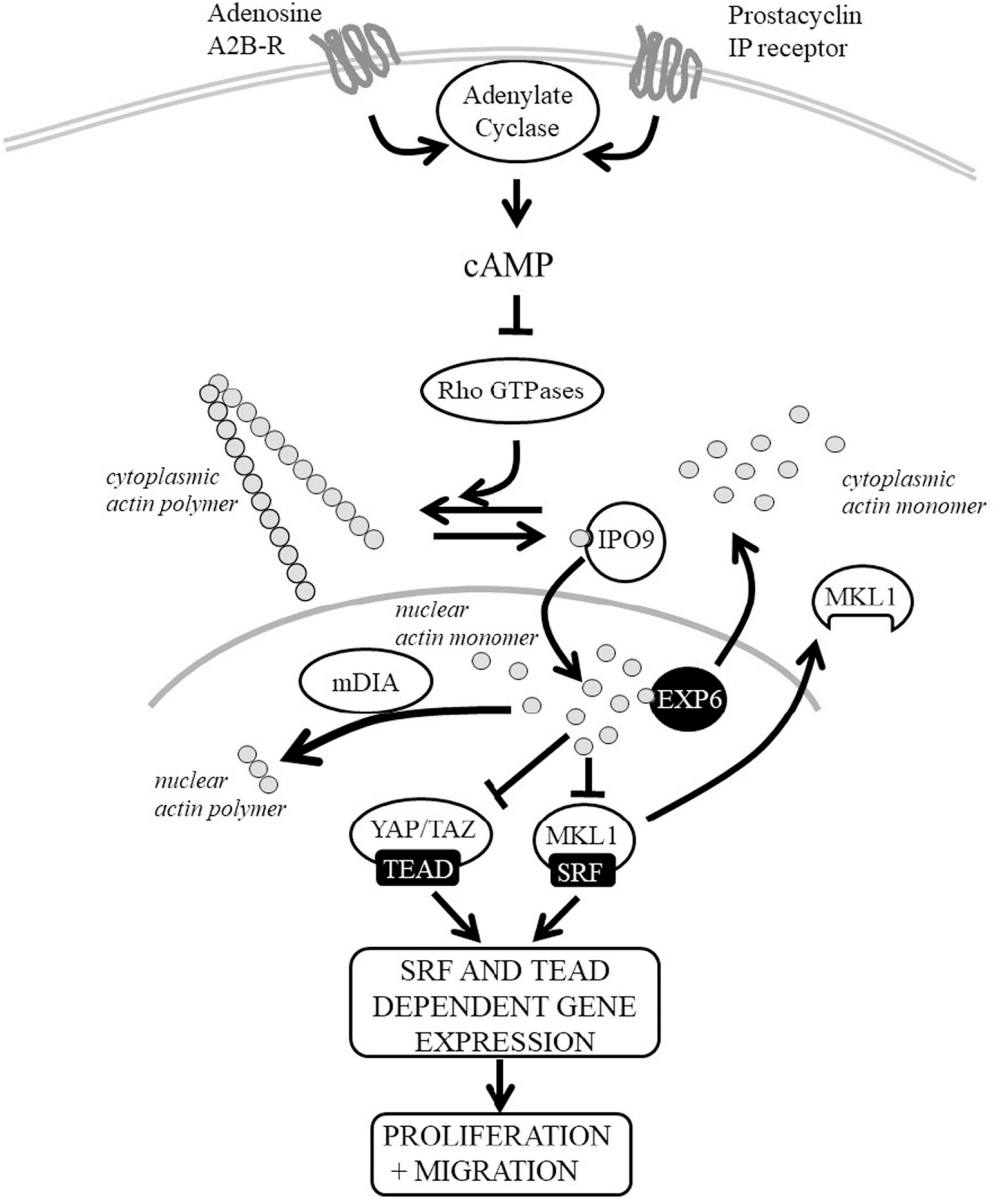
Proposed model of nuclear actin dependent regulation of YAP/TAZ-TEAD and MKL-SRF-dependent gene expression Activation of adenylyl cyclase in response to G_s_-coupled GPCR agonists increases intracellular cAMP levels. Elevated cAMP inhibits RhoGTPase-dependent polymerisation of cytosolic actin, thus increasing the availability of cytosolic actin monomer. Actin monomer is transported into the nucleus in association with importin-9 (IPO9). Nuclear actin monomer levels are also regulated by nuclear export, via exportin-6 (XPO6) or via their polymerisation, which is promoted by nuclear formins such a mDIA. Nuclear actin inhibits SRF activity, at least in part via nuclear export of MKL1. Nuclear actin inhibits YAP-TEAD activity, independently of YAP nuclear export. Nuclear actin-dependent inhibition of MKL-SRF and YAP/TAZ-TEAD reduces expression of genes required for efficient proliferation and migration.

In this study, we present evidence that nuclear actin plays a major inhibitory role in regulating proliferation and migration of VSMCs. We show that nuclear actin monomer levels rapidly increase in response to multiple cAMP elevating stimuli, including activation of the adenosine A2B and the prostacyclin receptors, which have widely documented anti-mitogenic and anti-migratory effects in these cells [23,35,36,53,56,64]. Increasing nuclear actin monomer levels via expression of NLS-Actin_R62D_, over expression of the actin importer, IPO9, or silencing of the actin exporter, XPO6, all significantly inhibited proliferation and migration. Moreover, preventing the cAMP mediated increase in nuclear actin, either by enhancing its nuclear export with XPO6 overexpression or by expressing nuclear localised constitutively-active mDIA, which has previously been shown to promote nuclear actin polymerisation [2], reversed the anti-mitogenic and anti-migratory effects of forskolin. Although forskolin can induce supraphysiological levels of intracellular cAMP in VSMCs [53]_,_ we demonstrate that XPO6-mediated reduction of nuclear actin monomer levels also reverses the anti-mitogenic and anti-migratory effects of BAY60-6583, an adenosine A2B-receptor agonist that induces physiological levels of cAMP in these cells [53]. Transcriptomic analysis of nuclear actin and cAMP regulated genes detected a large overlap in genes repressed by both signals, with repressed genes enriched in functions associated with proliferation and migration. Importantly, transcription factor enrichment analysis of the promoters of these genes identified enrichment of binding elements for SRF and TEAD, suggesting a functional role for these transcription factors in mediating the effects of intranuclear actin. Consistent with this, we show that nuclear actin or cAMP inhibited SRF and TEAD activity and inhibited expression of SRF and TEAD target genes. Moreover, we show that the inhibitory effects of cAMP on SRF and TEAD-dependent gene expression are mediated via increased nuclear actin. Furthermore, inhibition of TEAD functionally underlies the anti-mitotic and anti-migratory effects of nuclear actin since constitutively active mutants of the TEAD co-factors YAP or TAZ completely rescued proliferation and migration in cAMP stimulated cells. Rescuing SRF activity via expression of a constitutively active MKL1 only partially rescued proliferation, suggesting a dominant role for the YAP/TAZ-TEAD pathway over MKL-SRF. The inhibitory effects of nuclear actin on proliferation and migration were mediated, at least in part via inhibition of the SRF and TEAD-target genes *Heyl, Zyxin* and *Pai1*. Taken together, these data demonstrate that intranuclear actin mediates the inhibition of proliferation and migration by physiological signals that activate cAMP signalling through inhibiting expression of SRF and TEAD-target genes. Our data elucidates a new mechanism (see Fig. 9) that links changes in intranuclear actin in response to physiological signals to changes in cell proliferation and migration.

The presence of actin within the nucleus was originally reported in the 1960s, although at the time this was not widely accepted, and its function was unknown [8,31,61]. More recently, reports have confirmed the existence of actin in the nucleus and characterised its role in the regulation of gene transcription, chromatin remodelling and cell differentiation [32]. Although its existence within the nucleus is now well established, very little is known about the physiological signals that control nuclear actin levels. In epithelial cells, interaction with extracellular matrix components such as Laminin-111 promotes nuclear export of actin via increasing XPO6 [18]. In rat peritoneal mast cells, ATP depletion is associated with an increase in nuclear actin levels, suggesting that nuclear actin may be involved in cellular stress responses [45]. Increased levels of nuclear actin have also been detected after phorbol ester stimulation of monocytic HL-60 cells, where it is implicated in transcriptional regulation of macrophage differentiation [66]. Here we demonstrate for the first time that physiological signals that elevate cAMP promote a rapid increase in nuclear actin monomer levels in VSMCs. We detected increases in nuclear actin monomer as early as 1 h after stimulation with the adenosine A2B-receptor agonist (BAY60-6583), which elevates intracellular cAMP [53]. This implies that increases in nuclear actin levels is an early event preceding the inhibitory effects of cAMP on proliferation and migration. Precisely how cAMP signalling results in an increase in nuclear actin levels is currently unknown. However, in VSMCs, cAMP signalling is known to rapidly inhibit RhoGTPase activity and increase the F:G actin ratio [5,53]. Changes in cytoplasmic actin monomer levels are known to quickly lead to changes in nuclear actin monomer levels [20] and is a rate limiting factor determining actin import [10]. Consistent with this, we observed an increase in nuclear actin monomer levels following latrunculin-B-mediated depolymerisation of cytoplasmic actin. Therefore, a rapid increase in cytoplasmic actin monomer is likely, at least in part, to underlie the increase in nuclear actin monomer in response to cAMP. Increased levels of intranuclear actin monomer can promote the formation of filaments of nuclear actin polymer [46]. However, our data suggest that it is the increase in monomer, rather than polymer, that is responsible for the anti-mitogenic and anti-migratory effects of cAMP. For example, expression of mDIA-CT reduces nuclear actin monomer levels by enhancing polymerisation within the nucleus and increases cell proliferation and migration. In contrast, elevated cAMP increases nuclear actin monomer levels but inhibits proliferation and migration.

The anti-mitogenic and anti-migratory properties of cAMP are well characterised [7,24,27,53,54] but the underlying mechanisms are not fully understood. Previous research has implicated inhibition of Rho GTPases and impaired actin polymerisation, which is associated with the acquisition of a characteristic stellate morphology, characterised by reduced cell spreading and loss of F-actin stress fibres [24]. These effects on the cytoplasmic actin cytoskeleton were assumed to be responsible for the anti-mitogenic and anti-migratory effects of cAMP since expression of constitutively active Rho GTPases could reverse the effects of cAMP on cell spreading, cytoplasmic actin polymerisation and ultimately proliferation and migration [5]. However, we now demonstrate that cAMP elevating stimuli also increase levels of actin monomer within the nucleus. It is noteworthy that one of the earliest reports of actin being present within the nucleus found that contact inhibition of fibroblast growth resulted in *“a significant increase in the intranuclear concentration of a protein which comigrates with Physarum nuclear actin”* [38], although the functional significance of this was unknown at the time. Our new data demonstrates that increased levels of nuclear actin monomer underlie the anti-mitogenic and anti-migratory effects of cAMP in VSMCs.

The anti-mitogenic effects of cAMP are known to be cell-type specific, which may in part reflect divergent effects of cAMP on cytoplasmic actin reorganisation in these cells [53]. For example, cAMP induces rapid loss of actin stress fibres and actin depolymerisation in some cell types, including fibroblasts [15], mesenchymal stem cells [69] and VSMC [5,34] [12], while other cell types, such as ECs [53], cAMP promotes actin polymerisation and formation of cortical actin stress fibres. Consistent with this, we observed opposing effects of cAMP on intranuclear actin monomer levels in VSMCs compared to ECs, being increased in VSMCs and decreased in ECs. This may simply mirror changes in cytoplasmic actin monomer availability that occurs in these cells after cAMP stimulation. Nevertheless, the function of intranuclear actin in these cells also appears to be cell-type specific. Over expression of NLS-Actin_R62D_ in ECs resulted in a small increase in proliferation and did not affect migration rate, in contrast to its inhibitory effects in VSMCs. Interestingly, nuclear actin appears to play a pro-mitogenic function in epithelial cells, where quiescence induced by culture on a laminin-111 is associated with reduced levels of nuclear actin and reduced gene transcription. In these cells, expression of NLS-Actin_R62D_ rescues their proliferation and restores transcription [18,57].

Our transcriptomic analysis detected both increased and decreased expression of many genes in response to elevated levels of nuclear actin, indicating that nuclear actin modulates specific gene regulatory mechanisms instead of globally effecting transcription per se. Our analysis of nuclear actin repressed genes detected enrichment of genes associated with proliferation and migration. Importantly, we detected a substantial overlap between genes repressed by elevated cAMP or nuclear actin, consistent with a model where cAMP represses genes required for proliferation by elevating intranuclear actin levels. Transcription factor enrichment analysis and GSEA of these repressed genes identified SRF and TEAD dependent gene signatures, indicating inhibition of SRF and TEAD underlies many of the transcriptional changes we detected. Inhibition of SRF and TEAD was confirmed by reporter gene analysis. Inhibition of SRF activity is consistent with data obtained in NIH 3T3 fibroblasts, where nuclear actin regulates MKL nuclear export and activity of MKL-SRF complexes [61]. An SRF gene signature is consistent with our previous demonstration that cAMP represses SRF activity in VSMCs [53], which also occurs, at least in part, via enhanced nuclear export of MKL1 and our observation reported here that nuclear actin also prevents accumulation of MKL1 in the nucleus and inhibits SRF activity. Interestingly, expression of constitutively active actin independent MKL1 (ΔN100-MKL), lacking the actin-binding RPEL domains, only partially reversed the inhibitory effects of nuclear actin on proliferation, implicating additional mechanisms. Nevertheless, ΔN100-MKL completely reversed the inhibition of migration by NLS-Actin_R62D,_ suggesting a more important role for MKL1 in the regulation of migration compared to proliferation. This is consistent with previously reported MKL1-linked gene signatures being associated with cell motility and cytoskeleton dynamics [17]. On the other hand, very little is known about the role of nuclear actin in the regulation of TEAD activity. Here we demonstrated inhibition of TEAD reporter activity and target gene expression by nuclear actin. Importantly, we found that active mutants of YAP or TAZ completely reversed the anti-mitogenic effects of nuclear actin, implying a major role for the YAP/TAZ-TEAD axis compared to the MKL1-SRF axis in mediating the growth inhibitory effects of intranuclear actin. These data highlight for the first time that inhibition of YAP/TAZ-TEAD is an important mechanism mediating the biological effects of nuclear actin. Although our data clearly demonstrates inhibition of TEAD activity by nuclear actin, the underlying mechanisms are unknown. Inhibition of TEAD activity was not associated with nuclear export of YAP or changes in YAP phosphorylation status, in contrast to cAMP or disruption of the cytoplasmic actin cytoskeleton, which did induce YAP export and phosphorylation [36]. This suggests that the nuclear YAP/TAZ-TEAD complex is susceptible to regulation by nuclear actin via a mechanism that is independent of changes in co-factor nuclear export of phosphorylation (See Fig. 9). This is model is consistent with recent data demonstrating that YAP constrained to the nucleus by XPO1 silencing remains sensitive to actomyosin disruption [16]. Recent reports have described reciprocal regulation between MKL-SRF and YAP-TEAD pathways [19] and enhancement of YAP-TEAD activity via direct interactions and formation of an MKL1-YAP-TEAD complex [33], raising the possibility that inhibition of TEAD activity by nuclear actin may occur as a result of actin sequestration of MKL1. However, our data does not support this model, instead pointing to an MKL1-independent repression of TEAD activity. Given the well characterised role of TEAD transcription factors in the regulation of cell proliferation and their dysregulation in hyper-proliferative pathologies and cancer, it is important that future research focusses on characterising these mechanisms further.

Although there has been great interest in the molecular functions of intranuclear actin in recent years, relatively little is known about the importance of nuclear actin dynamics in the regulation of cell behaviour. Here we describe a novel role for nuclear actin monomer in the regulation of cell proliferation and migration of vascular smooth muscle cells. We show for the first time that elevated nuclear actin levels inhibit cell proliferation and migration of these cells. We show that this is mediated, at least in part, via inhibition of the Hippo-pathway target transcription factor complex YAP/TAZ-TEAD. We also show that this novel mechanisms accounts for the anti-mitogenic and anti-migratory effects of physiological signals that elevate cAMP.

## 4 Materials and methods

### 4.1 Reagents

All chemicals were obtained from Sigma unless otherwise stated. Antibodies to YAP and Lamin A/C (#4777) were from Cell Signalling Technologies. Antibody to GAPDH (MAB374) was from Millipore. Antibodies to YAP (#140745), YAP phospho-serine-127 (#4911), YAP phospho-serine-397 (#13619) and FLAG-tag (#14793) were from Cell Signalling Technologies. Antibody to XPO6 (ab72333) was from Abcam. Silencer Select siRNA targeting rat XPO6 (s147550) and non-targeting negative control (#4404020) were from Thermo Fisher Scientific.

### 4.2 Smooth muscle and endothelial cell culture

Male Sprague Dawley rats were killed by cervical dislocation in accordance with schedule 1 of the U.K. Animals (Scientific Procedures) Act 1986 and Directive 2010/63/EU of the European Parliament and with the approval of the University of Bristol. Methods used to culture VSMCs and ECs are described in detail in the Supplement. Human aortic VSMCs (HuVSMCs) and human umbilical endothelial cells (HUVC) at passage 2–8 were purchased from Promocell. All experiments were replicated for the number of time shown in the text and figures using different batches of cells that were prepared from different animals/donors. Cultures of rat aortic VSMCs (RaVSMCs) were prepared as previously described [22] and cultured in Dulbecco's Modified Eagle's Medium (DMEM) supplemented with 10% foetal bovine serum, 100 U/ml penicillin/streptomycin and 2.5 mM l-glutamine. Human VSMC were cultured in human smooth muscle cell growth medium 2 (Promocell), supplemented with the supplement pack (Promocell), 100 U/ml penicillin/streptomycin and 2.5 mM l-glutamine. HUVEC were cultured in Endothelial Cell Growth Medium 2 supplemented with the supplied supplement pack (Promocell), 100 U/ml penicillin/streptomycin and 2.5 mM l-glutamine.

### 4.3 Real-time scratch wound migration assays

Real-time analysis of migration was performed using an IncuCyte® ZOOM live cell imaging system (Essen BioScience) according to the manufacturer's instructions. Briefly, cells were seeded (2 × 10^4^ cells/well for RaVSMCs, 1 × 10^4^ cells/well for HuVSMCs and 2 × 10^4^ cells/well for HUVECs) into ImageLock-96 well plates. Wells were scratched using a WoundMaker® tool and phase contrast images of cell migration into the wounded area acquired hourly for 24 h. Relative wound confluence was calculated using the Cell Migration Image analysis module of the IncuCyte ® ZOOM software.

### 4.4 Quantitative RT-PCR and western blotting

Quantification of mRNA and protein levels was performed by RT-qPCR and western blotting respectively, as described previously [24]. Total RNA was extracted using Ambion Pure-Link kits (Thermo Fisher) and was reverse transcribed using QuantiNova RT kit (Qiagen) and random primers. Quantitative PCR was performed using Roche SYBR Green using a Qiagen Roto-Gene Q PCR machine (20’@95 °C;20’@62 °C;20’@72 °C). Primers sequences are described in Supplement Table 1. Data were normalised to total amount of RNA. Western blots were performed using a Mini-Protean II system. Proteins were transferred to PVDF membrane using a semi-dry Turbo blotter system (Bio-Rad) and detected using ECL and a digital ChemiDoc imaging system (Bio-Rad). Phos-tag gels were prepared containing 100 μM Phos-tag acrylamide and 20 μM MnCl_2_ according to the manufacturer's instructions (Alpha Laboratories).

### 4.5 Plasmids, siRNA and adenoviral vectors

Replication deficient adenoviral vectors were generated by cloning cDNAs into the multiple cloning site of the pDC515 adenovirus shuttle vector (Microbix) as previously described [36]. Adenovirus expressing FLAG-tagged actin containing a nuclear localisation signal and an R62D mutation to prevent polymerisation was generated by cloning FLAG-NLS-β-ACTIN-_R62D_ cDNA into the EcoR1 and Nhe1 sites of plasmid pDC515 followed by homologous recombination with adenovirus genome vector pBHG-frt in HEK293 cells. Adenovirus (Ad:mDIA-CT) expressing constitutively active nuclear mouse mDIA was made by subcloning the F1F2+C fragment of mDIA1 from pEF-mDIA-F1F2+C plasmid (a generous gift from John Copeland and described previously [2]) into the BamH1 and Sal1 sites of pDC515. Adenovirus expressing mouse Importin-9 (IPO9) was generated by PCR amplifying the IPO9 cDNA from MGC cDNA clone BC098508 and cloning into the Nhe1 and Sal1 sites of pDC515. Adenovirus expressing human Exportin-6 (XPO6) was amplified by PCR and cloned into the Nhe1 and BamH1 sites of plasmid pDC515. Plasmid TEAD-LUC expressing firefly luciferase under the control of a synthetic promoter containing eight copies of a consensus TEAD binding element was obtained from Addgene (#34615) and has been described previously [36] [13]. Plasmid SRE-LUC expressing firefly luciferase under the control of a synthetic promoter containing five SRE elements was purchased from Agilent. All Silencer Select siRNAs were purchased from Invitrogen Life Technologies.

### 4.6 Transient transfection and reporter gene assays

TEAD and SRF reporter gene activity was determined by quantifying the cellular luciferase reporter activity in cells transfected with TEAD-LUC or SRE-LUC. Plasmid transfection was performed by electroporation of 1 × 10^6^ cells with 5 μg of plasmid DNA using an Amaxa Nucleofector-1.5 (program A-033). For gene silencing, cells were transfected with 100 pmoles of Silencer Select siRNA (Life Technologies). At the indicated times post transfections, cell lysates were prepared in ice cold Cell Culture Lysis (CLB) buffer (Promega) and assayed for firefly luciferase activity using the luciferase assay system (Promega) and a Glomax Discover luminometer (Promega) according to the manufacturer's instructions.

### 4.7 Quantification of intranuclear actin using DNAse1 staining

Levels of intranuclear actin were detected by staining fixed cells with a DNAse1-Alexa Fluor-594 conjugate, essentially as previously described [57] [37]. Cells were fixed for 10 min at room temperature in 4% methanol-free formaldehyde. Cells were permeabilised with 0.1% Triton-X-100 and blocked in 5% bovine serum albumin for 20 min at room temperature followed by incubation with 9 μg/ml of DNAse1-Alexa Fluor-594 for 20 min at room temperature. Cells were washes in PBS and the nuclei counter stained with Hoechst 33342. Cells were imaged using a Leica confocal microscope. The nuclear DNAse1-Alexa Fluor-594 fluorescent signal was quantified in *Z*-slice images passing through the nuclei using an automated ImageJ script.

### 4.8 RNA-seq and bioinformatics

Total RNA was extracted from VSMC and EC using the PureLink RNA Mini Kit (Ambion) as per manufacturer's instructions. For RNA-sequencing, an additional on column DNase1 treatment step was included to assure highly pure RNA without genomic DNA contamination. RNA was then quantified using Nanodrop spectrophotometer technology (LabTech; model number: ND-1000). RNA concentration was obtained by measuring absorbance at 260 nm and 260/230 and 260/280 ratios determined the extent of ethanol and protein contamination, respectively. Only samples with sufficient purity were used for subsequent experiments. RNA-seq libraries were prepared using Illumina TruSeq stranded total RNA sample preparation kits including a ribodepletion step. Libraries were sequenced (100 bp paired end reads) using an Illumina HiSeq next generation sequencer. Raw reads were assessed for quality using the FastQC tool and trimmed as necessary using Cutadapt (a Python Command Line Tool, Dortmund, Germany). Trimmed reads were then aligned using HISAT2 and assembled using StringTie (http://ccb.jhu.edu/software.shtml). Raw count data was produced from the aligned reads using featureCounts (an R package, http://bioinf.wehi.edu.au/featureCounts/), and then differential expression analysis using edgeR (*Empirical Analysis of Digital Gene Expression Data in R*, https://bioconductor.org/packages/release/bioc/html/edgeR.html). Enrichment of transcription factor binding elements in gene promoters was performed using the online oPOSSUMv3.0 tool (http://opossum.cisreg.ca/oPOSSUM3/) [60]. Consensus binding elements for TEAD (TEAD1:MA0090.1; TEAD2: MA1121.2;TEAD3:MA0808.1; TEAD4:MA0809.1) in the 10kbp upstream of the transcription start site of regulated genes were identified using FIMO (http://meme-suite.org/tools/fimo) [3]. Gene ontology enrichment analysis was performed using the DAVID bioinformatics resource (https://david.ncifcrf.gov/) [9]. Gene Set Enrichment analysis was performed using R packages clusterProfiler [68] and fgsea [51] with 1000 permutations on pre-ranked gene lists generated using R package DESeq2 [41]. As necessary human homologues for rat gene IDs were obtained using the BiomaRt R package [14]. Gene sets were obtained from the Broad Institute Molecular Signatures Database [59] [39]: Hallmark [40] and C3, Transcription Factor Targets [65]; Zhao et al. supplementary Table 2, “Common Genes” [70]; Ensault et al., supplementary Table 5, (Bind SRF, serum-inducible, responsive to SRF-linked signals) [17]. TEAD ChIP (ENCODE) lists were generated by overlapping peaks for Tead4 ChIP from cell lines HepG2, K562, A549 and MCF7. SRF ChIP (ENCODE) lists were generated by overlapping peaks for SRF ChIP from cell lines GM12878, HepG2, K562, MCF7 and Hct11. Peaks were assigned to genes using R package ChIPpeakAnno [71].

### 4.9 Statistical analysis

After testing for Gaussian distribution, statistical analysis was performed using two-way ANOVA, one-way ANOVA with Student-Newman-Keuls post-test or where appropriate a paired student's t-test, as indicated. *Indicates p < 0.05, **indicates p < 0.01, ***indicates p < 0.001.

## Declaration of competing interest

The authors declare that they have no known competing financial interests or personal relationships that could have appeared to influence the work reported in this paper.

## Appendix A Supplementary data

Supplementary material Image 1

Supplementary figures and table. Image 2
