# Supplementary figures and images for: Nuclear actin regulates cell proliferation and migration via inhibition of SRF and TEAD
Source: Biochim Biophys Acta Mol Cell Res. 2020 Jul;1867(7):118691. doi: 10.1016/j.bbamcr.2020.118691 (PMC7262588; doi:10.1016/j.bbamcr.2020.118691)

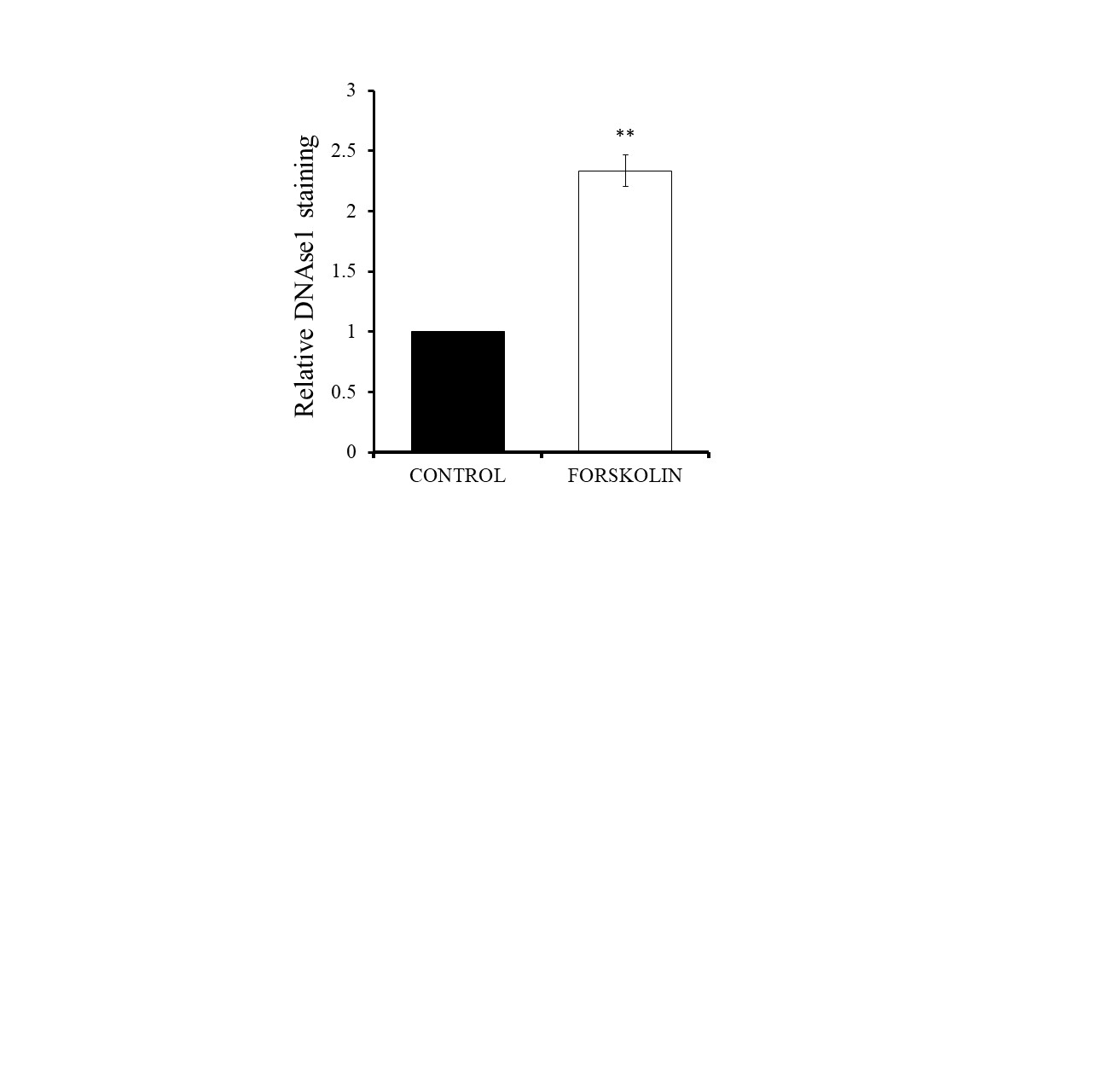

Supplement: Supplementary file 2 — Supplementary figures and table. [file mmc2.zip › Supplement figure 1_202005001822762432.JPG]

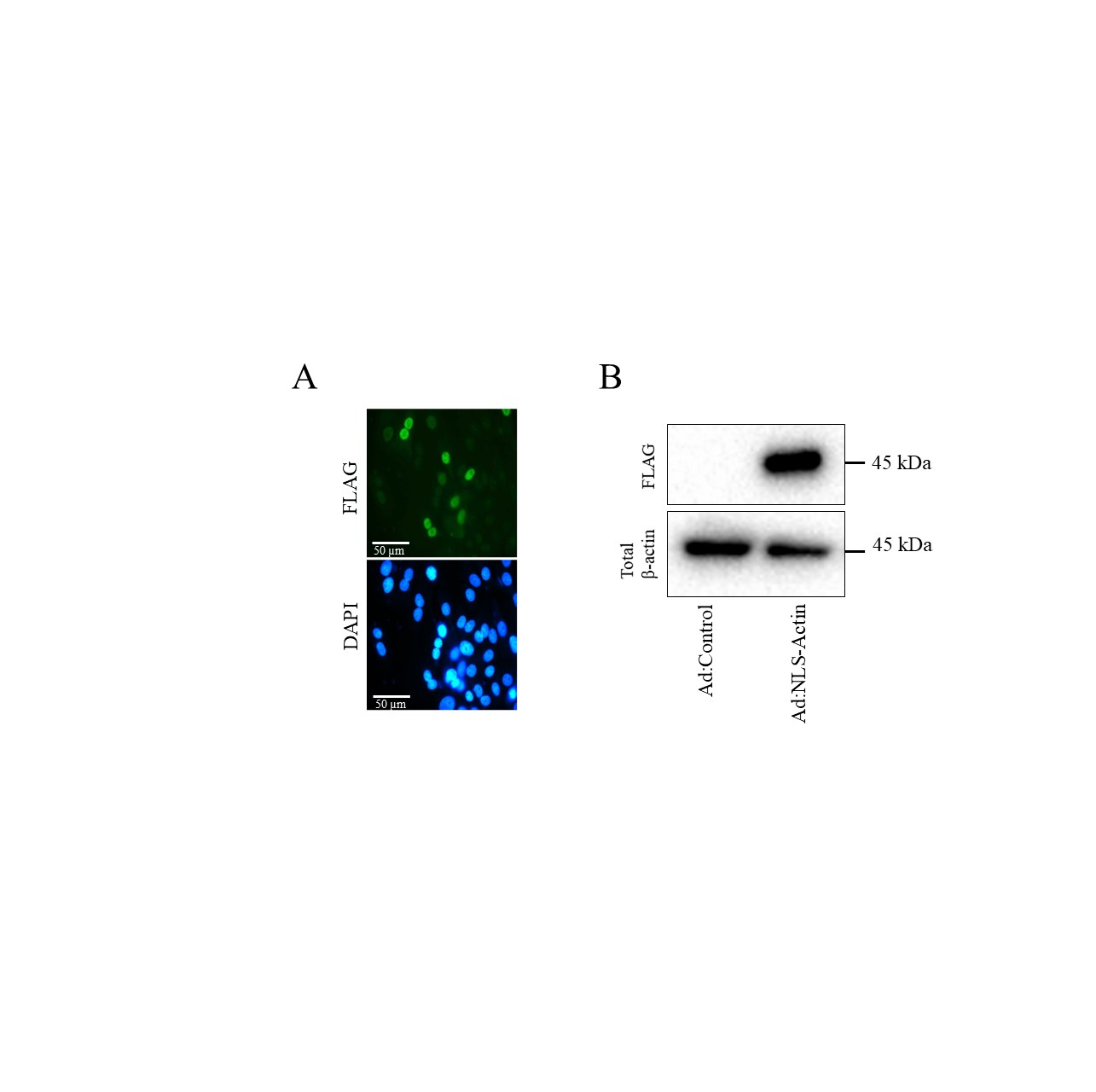

Supplement: Supplementary file 2 — Supplementary figures and table. [file mmc2.zip › Supplement figure 2_202005001822762433.JPG]

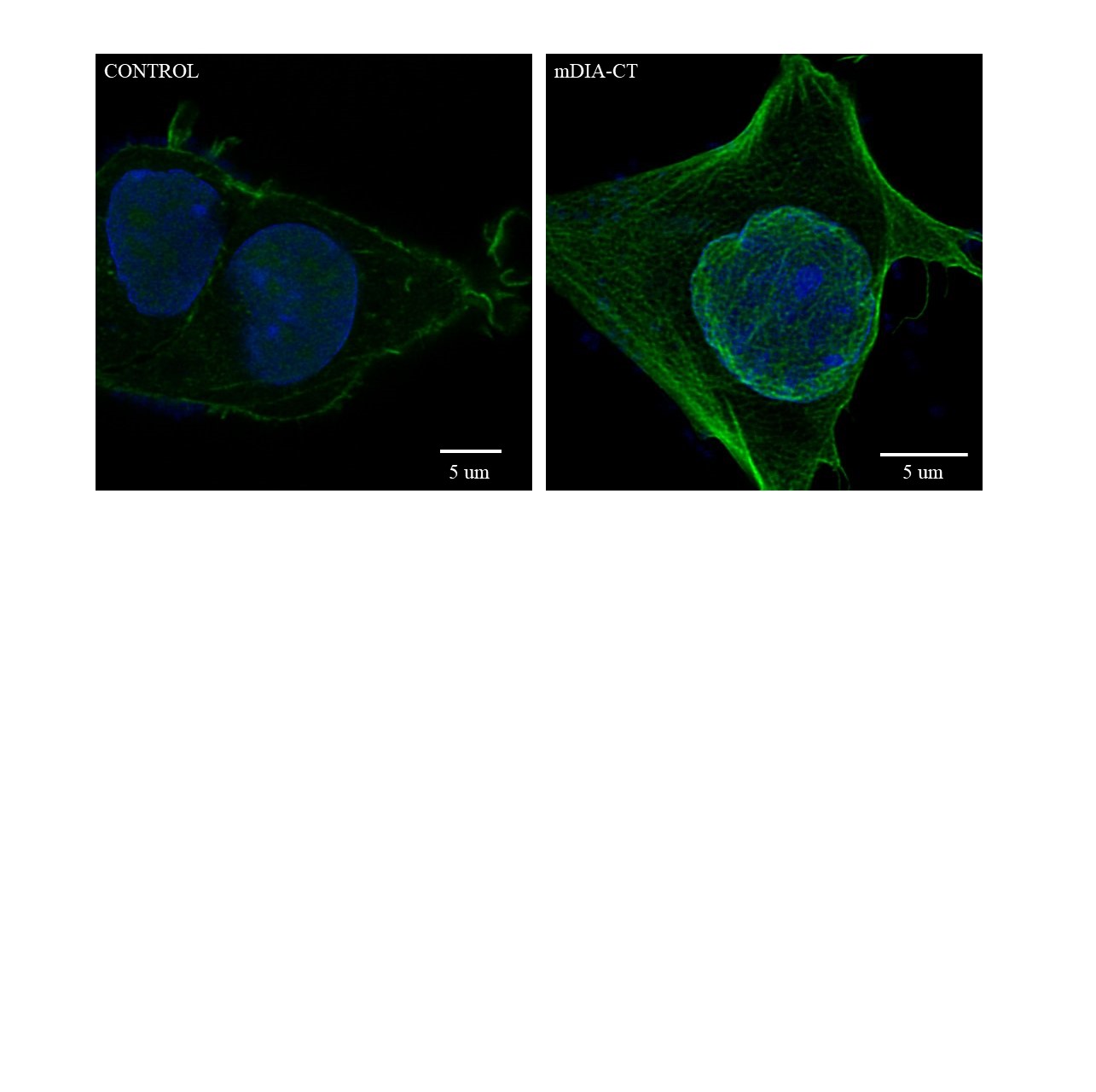

Supplement: Supplementary file 2 — Supplementary figures and table. [file mmc2.zip › Supplement figure 3_202005001822762434.JPG]

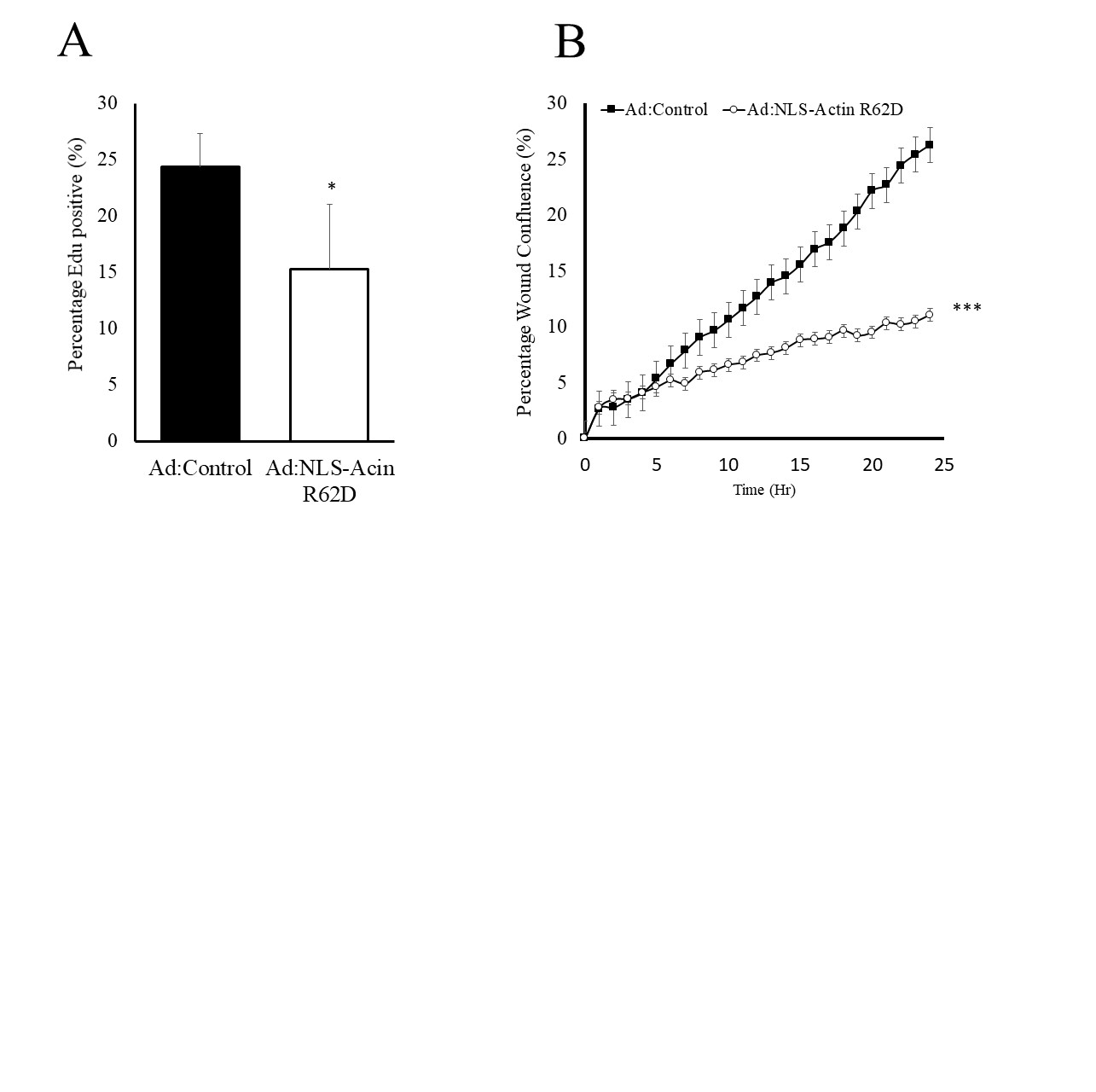

Supplement: Supplementary file 2 — Supplementary figures and table. [file mmc2.zip › Supplement figure 4_202005001822762435.JPG]

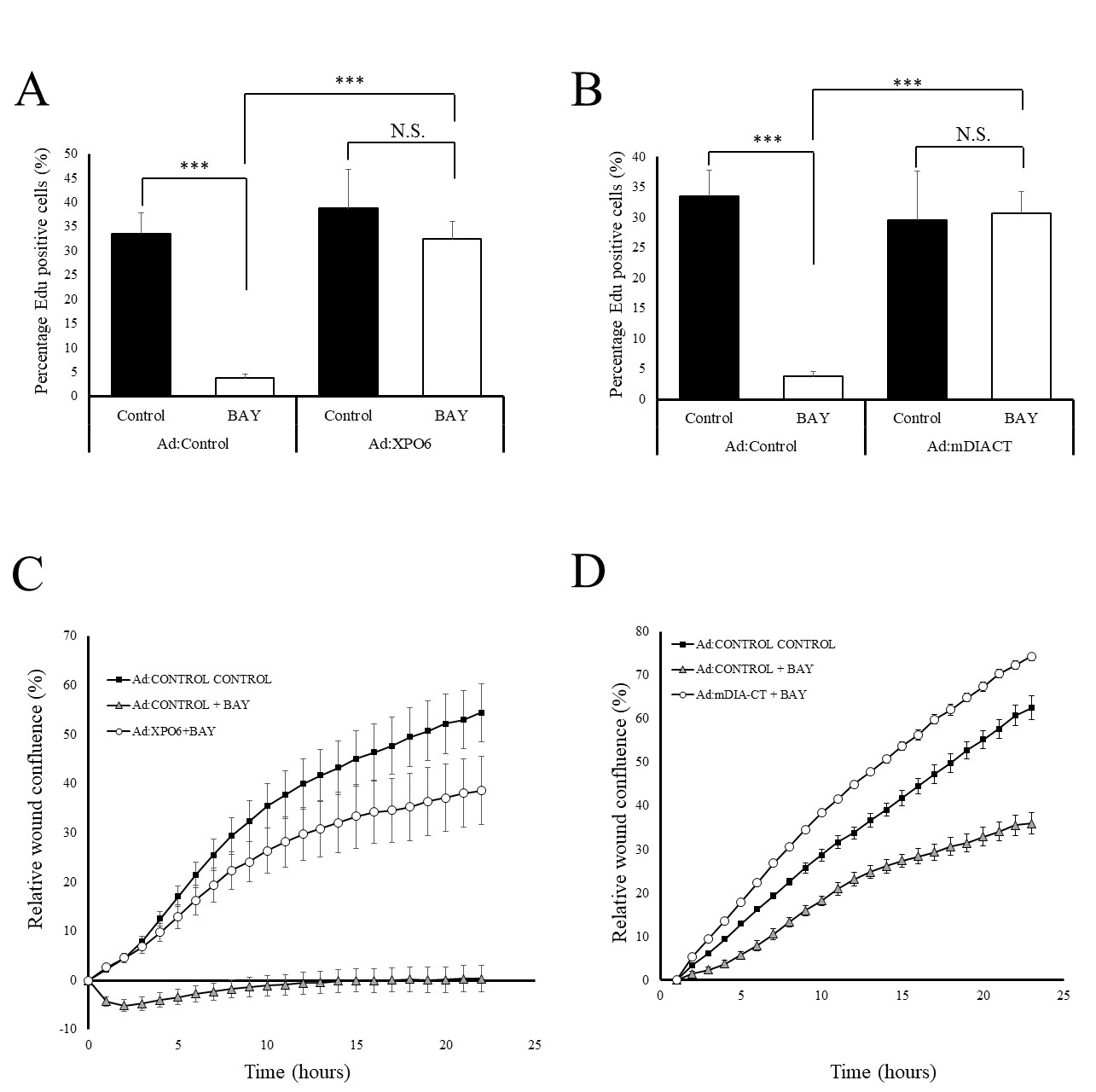

Supplement: Supplementary file 2 — Supplementary figures and table. [file mmc2.zip › Supplement figure 5_202005001822762436.JPG]

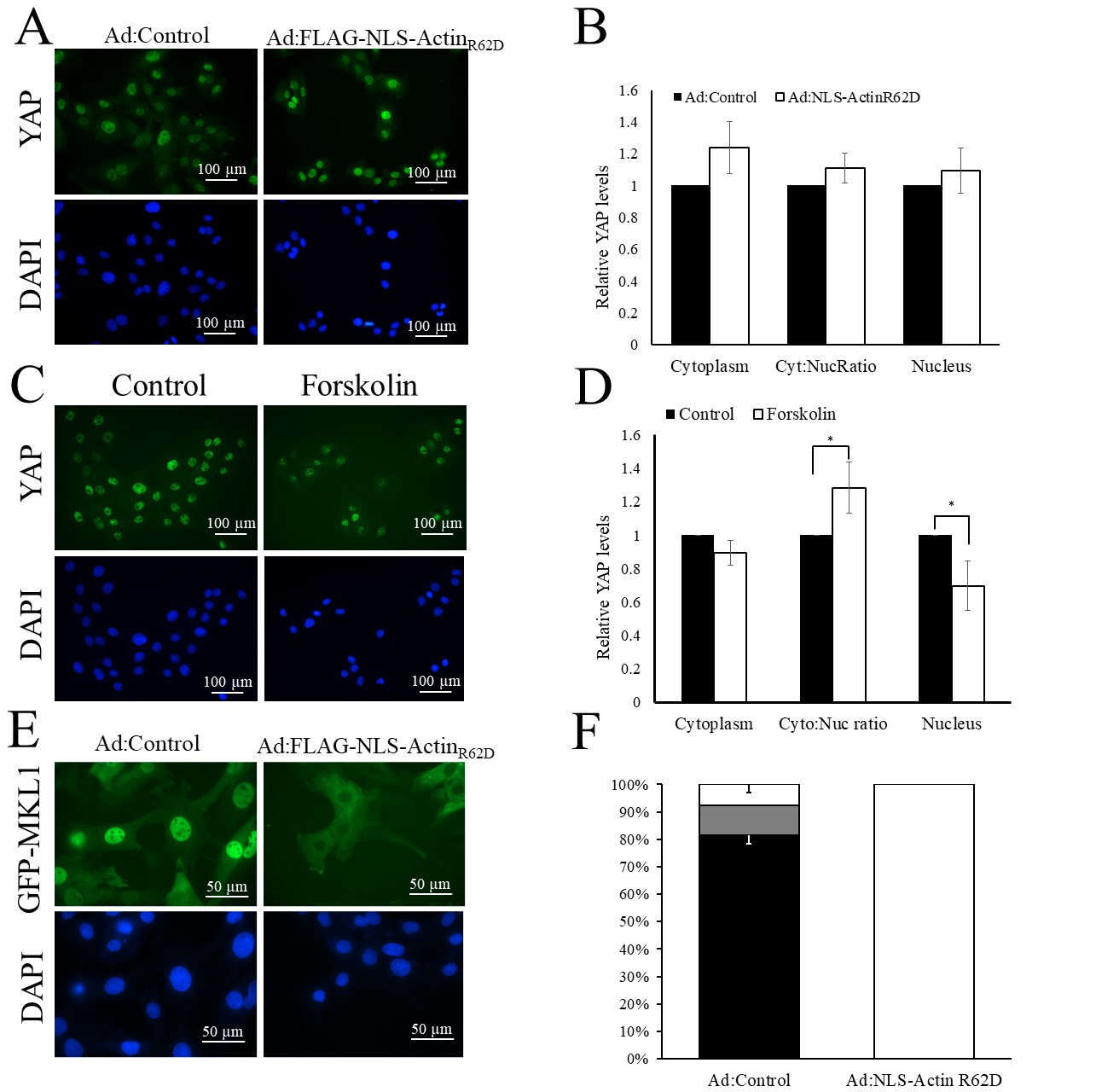

Supplement: Supplementary file 2 — Supplementary figures and table. [file mmc2.zip › Supplement figure 6_202005001822762437.JPG]

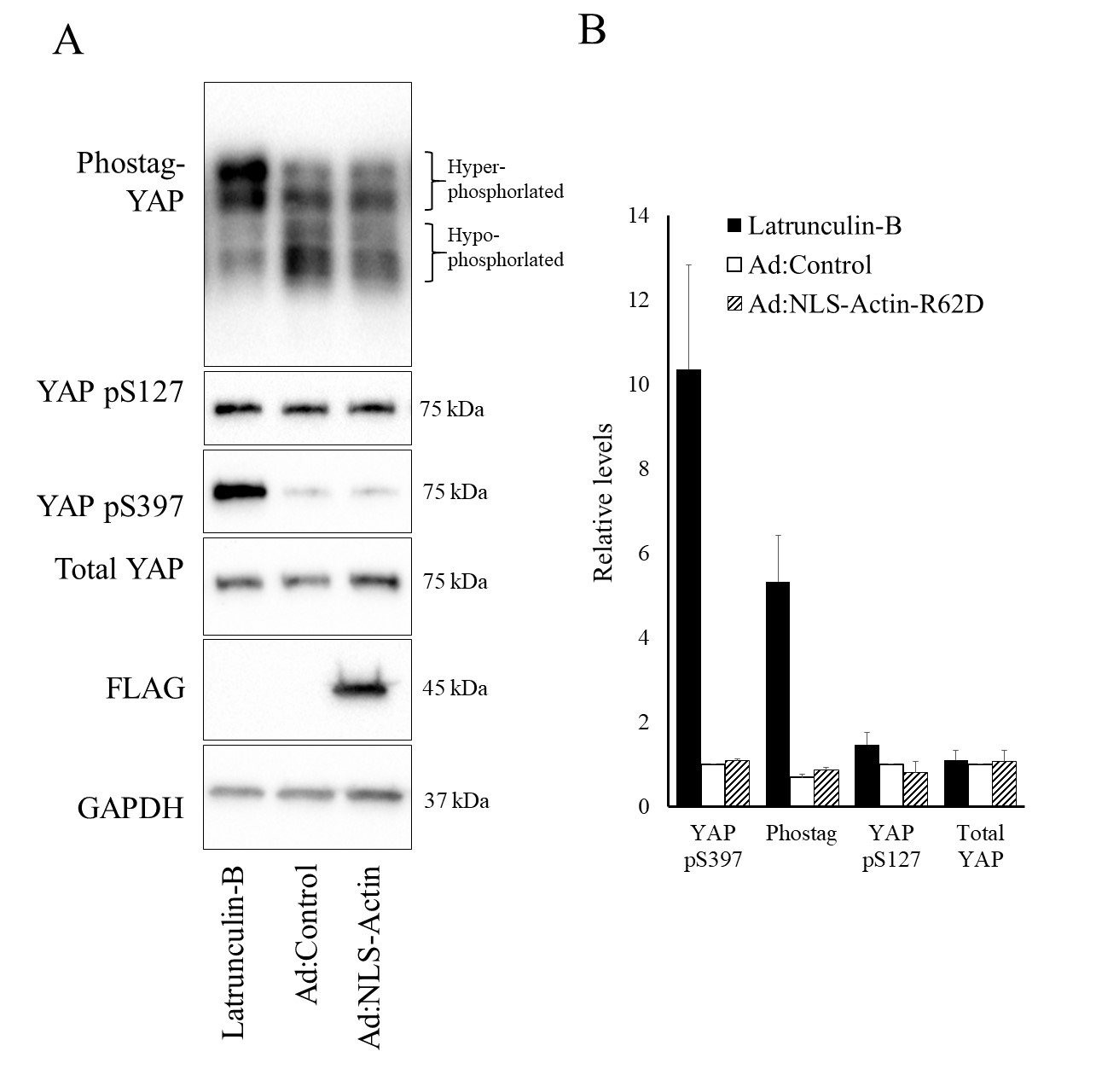

Supplement: Supplementary file 2 — Supplementary figures and table. [file mmc2.zip › Supplement figure 7_202005001822762438.JPG]

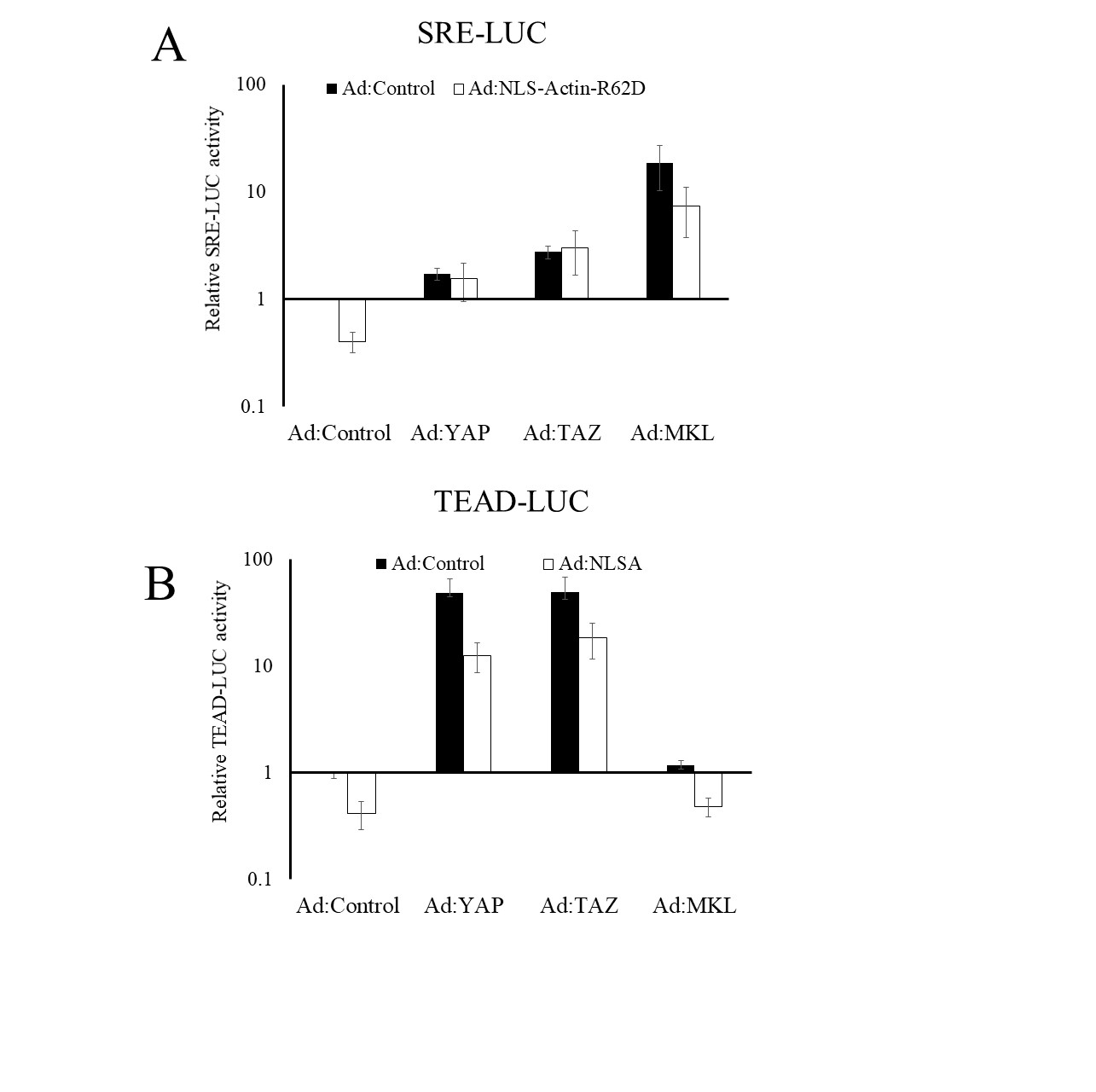

Supplement: Supplementary file 2 — Supplementary figures and table. [file mmc2.zip › Supplement figure 8_202005001822762439.JPG]

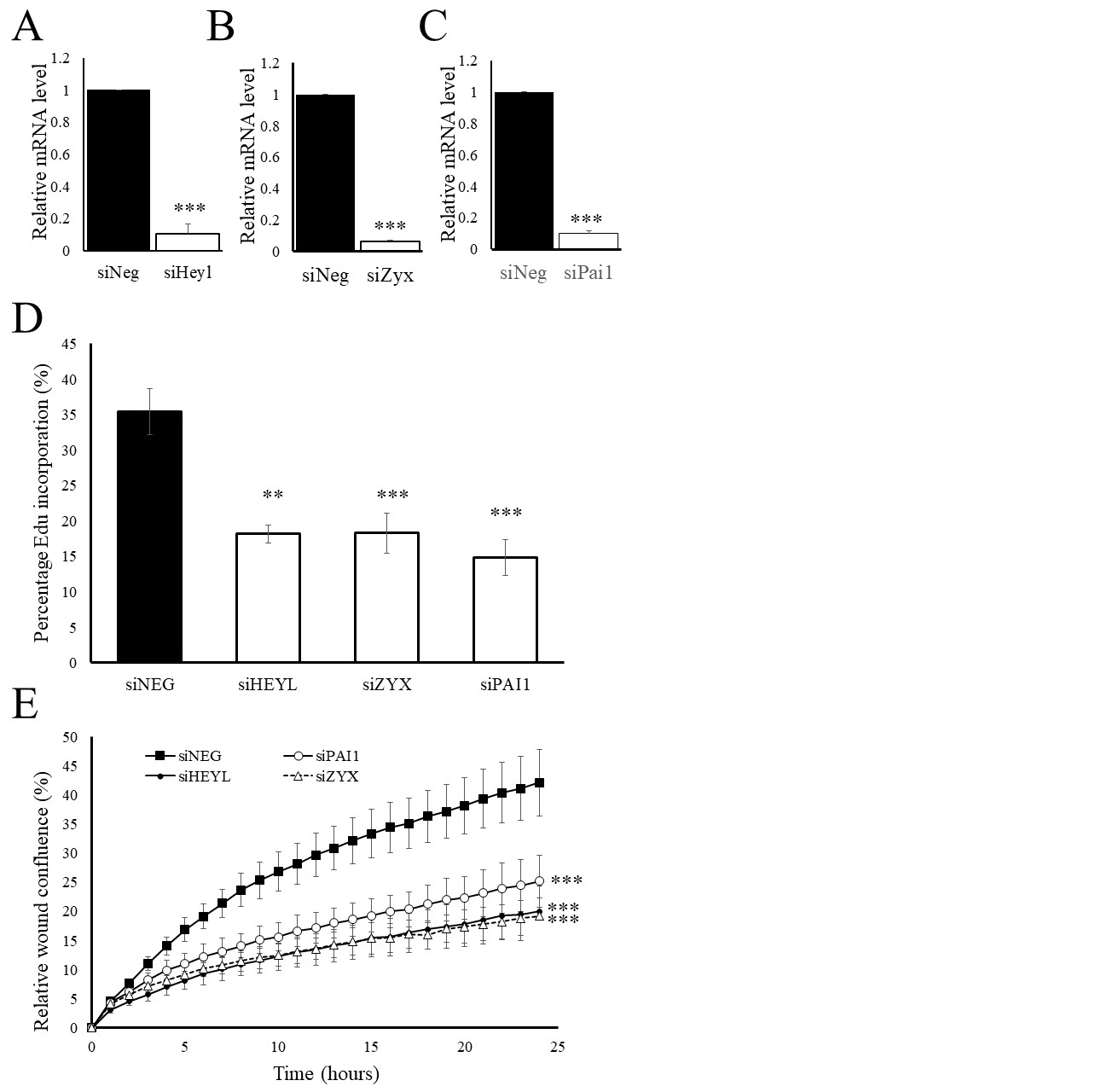

Supplement: Supplementary file 2 — Supplementary figures and table. [file mmc2.zip › Supplement figure 9_202005001822762441.JPG]

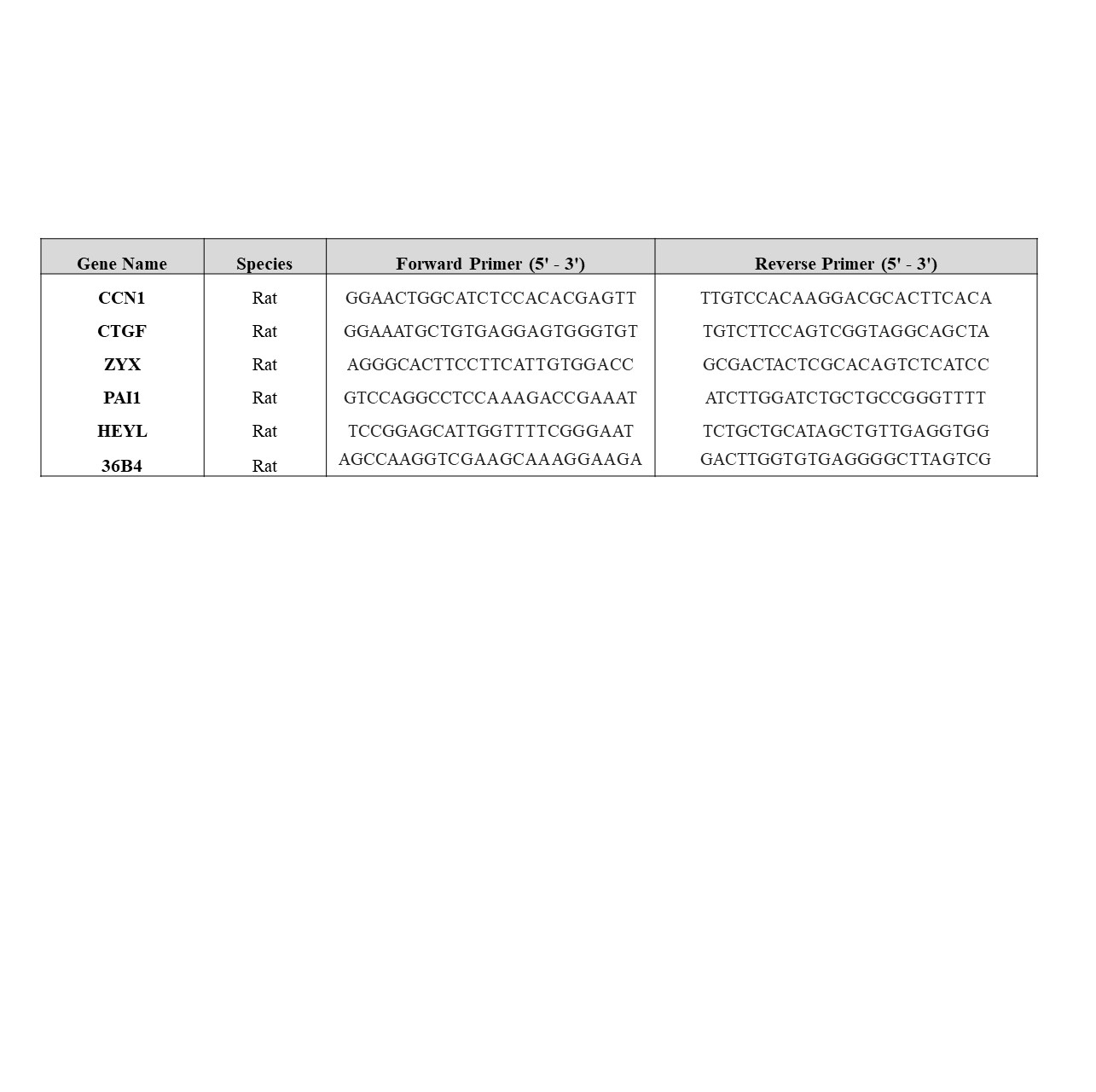

Supplement: Supplementary file 2 — Supplementary figures and table. [file mmc2.zip › Supplement table 1_202005001822762443.JPG]
